# CAMML with the Integration of Marker Proteins (ChIMP)

**DOI:** 10.1093/bioinformatics/btac674

**Published:** 2022-10-10

**Authors:** Courtney Schiebout, H Robert Frost

**Affiliations:** Department of Biomedical Data Science, Dartmouth College, Hanover, NH 03755, USA; Department of Biomedical Data Science, Dartmouth College, Hanover, NH 03755, USA

## Abstract

**Motivation:**

Cell typing is a critical task in the analysis of single-cell data, particularly when studying complex diseased tissues. Unfortunately, the sparsity and noise of single-cell data make accurate cell typing of individual cells difficult. To address these challenges, we previously developed the CAMML method for multi-label cell typing of single-cell RNA-sequencing (scRNA-seq) data. CAMML uses weighted gene sets to score each profiled cell for multiple potential cell types. While CAMML outperforms other scRNA-seq cell typing techniques, it only leverages transcriptomic data so cannot take advantage of newer multi-omic single-cell assays that jointly profile gene expression and protein abundance (e.g. joint scRNA-seq/CITE-seq).

**Results:**

We developed the CAMML with the Integration of Marker Proteins (ChIMP) method to support multi-label cell typing of individual cells jointly profiled via scRNA-seq and CITE-seq. ChIMP combines cell type scores computed on scRNA-seq data via the CAMML approach with discretized CITE-seq measurements for cell type marker proteins. The multi-omic cell type scores generated by ChIMP allow researchers to more precisely and conservatively cell type joint scRNA-seq/CITE-seq data.

**Availability and implementation:**

An implementation of this work is available on CRAN at https://cran.r-project.org/web/packages/CAMML/.

**Supplementary information:**

Supplementary data are available at *Bioinformatics* online.

## 1 Introduction

The immune cells present in tissues have important implications for health and function, particularly in diseased states (Azizi *et al.*, 2018; Hanahan and Coussens, 2012). Tumor-infiltrating immune cells are an important example, with immune cell presence and phenotype being key indicators of disease outlook and prognosis (Azizi *et al.*, 2018; Davidson *et al.*, 2020; Qiu *et al.*, 2018; Wagner *et al.*, 2019). In particular, the polarization state of macrophages and the presence and phenotype of T cells can predict the severity of tumor immune evasion and its downstream effects on tumor growth (Li *et al.*, 2019; Wagner *et al.*, 2019; Zhang *et al.*, 2019a). Thus, identifying infiltrating immune cells and characterizing their cell (sub)type and phenotype is a critical aspect of cancer research. For the analysis of bulk tissue data, e.g. bulk RNA-sequencing (RNA-seq), a common approach involves the application of deconvolution methods [e.g. CIBERSORT (Newman *et al.*, 2015), DeconRNAseq (Gong and Szustakowski, 2013)] to estimate the proportions of each cell type in the tissue. However, bulk tissue analysis only provides estimates of cell type proportions, requires prior knowledge of the cell types present in the tissue and does not work well for small cell populations or genes with low expression (Chen *et al.*, 2019; Li and Wang, 2021). These limitations inhibit bulk devolution methods from accurately detecting all cell types in a tissue without bias toward the phenotypes that are most highly expressed.

In order to reduce this lack of granularity, utilization of single-cell RNA-seq (scRNA-seq) has become increasingly popular for characterizing tissues (Azizi *et al.*, 2018; Hay *et al.*, 2018; Tang *et al.*, 2009; Wagner *et al.*, 2019). This allows each cell’s individual transcriptome to be analyzed independently of the other cells present in the tissue, enabling smaller signals and cell populations to be detected. However, this approach is not without limitations, with noise and sparsity being key challenges for the scRNA-seq analysis (Haque *et al.*, 2017; Kolodziejczyk *et al.*, 2015). To overcome these issues, cell typing of scRNA-seq data is often performed at the cluster level, rather than for single cells (Diaz-Mejia *et al.*, 2019; Kiselev *et al.*, 2019; Tirosh *et al.*, 2016). Specifically, it is common practice for investigators to cluster scRNA-seq data, determine the most differentially expressed (DE) genes present in each cluster, and then manually deduce the most likely cell type identity of each cluster based on the DE genes (Kiselev *et al.*, 2019). While this method leverages known biological context to identify cell types, it makes the assumption that all cells within a cluster have the same cell type, which is often not the case. Furthermore, given that cells are clustered by an unsupervised algorithm that considers all expressed genes, cells that are phenotypically similar may be assigned the same cell type even when the underlying cell identities are distinct (e.g. cytotoxic NK and T cells) (Kiselev *et al.*, 2019; Satija *et al.*, 2015). Cell typing at the cluster level can therefore lead to a high rate of misclassification, especially among phenotypically similar cell types.

Methods that assign cell types to individual cells, and thus do not assume cluster homogeneity, have been developed in response to this issue (Aran *et al.*, 2019; de Kanter *et al.*, 2019). However, these methods were designed to identify just a single-cell type for each cell, despite growing evidence that many cell types occur on a continuum rather than in discrete categories (Li *et al.*, 2019; Zhang *et al.*, 2019a). To address this limitation, we developed cell typing using variance Adjusted Mahalanobis distances with Multi-Labeling (CAMML), a multi-label scRNA-seq cell typing method that utilizes weighted cell type gene sets to score cells for their most likely identities (Schiebout and Frost, 2022). We found that CAMML achieved classification performance that was equal to or superior to existing methods with the added benefit of characterizing cells whose phenotype is on a spectrum (Schiebout and Frost, 2022). These features allow CAMML to better capture the underlying biology of complex cell populations where there is phenotypic overlap between cell types. Although CAMML provides a number of advantages relative to existing scRNA-seq cell typing techniques, it only utilizes transcriptomics data for cell type estimation so is unable to fully leverage the information generated by new multi-omic single-cell assays.

Advances in single-cell profiling techniques now allow investigators to measure multiple omics modalities on each cell. One such modality being employed in combination with scRNA-seq is Cellular Indexing of Transcriptomes and Epitopes by sequencing (CITE-seq), which quantifies the abundance of cell surface markers (Stoeckius *et al.*, 2017). This allows cell type-relevant protein markers on the surface of cells to be identified and quantified at a single-cell level (Stoeckius *et al.*, 2017). Given that cells are often identified by their surface markers, this technology is inherently informative for cell typing of single-cell data. However, very few markers can be quantified with CITE-seq (only a few hundred surface proteins at most versus the tens of thousands of genes scRNA-seq can measure) and CITE-seq often suffers from notable background noise (Mulè *et al.*, 2022; Stoeckius *et al.*, 2017). In order to overcome both scRNA-seq and CITE-seq’s limitations for cell typing, we integrated both modalities to create CAMML with the Integration of Marker Proteins (ChIMP), an extension of CAMML that allows users to perform conservative cell typing of joint CITE-seq/scRNA-seq data.

## 2 Materials and methods

### 2.1 Single-cell data sources and processing pipeline

Data were accessed from a study by Lawlor *et al.* (2021) where peripheral blood mononuclear cells (PBMCs) were isolated from healthy individuals and split into two samples. One sample was sorted with flow cytometry, and the other was analyzed with joint scRNA-seq and CITE-seq. Any cells labeled by the original article as multiplets were removed prior to further analysis (Lawlor *et al.*, 2021). Only cells with at least 1000 genes, of which no more than 5% were mitochondrial, were kept for analysis. Furthermore, given the significant volume of cells present in the data, any genes present in fewer than 100 cells were removed in order to reduce noise and computational load. The CITE-seq was merged following the filtering of the scRNA-seq data but no specific CITE-seq filtering steps were applied. The scRNA-seq was normalized using the Seurat log-normalization method (i.e. a log transformation was applied to the sum of a pseudo-count of 1 and the original count divided by the library size and multiplied by a scale factor of 10 000) and the CITE-seq was normalized by center log ratio transformation and both omics modalities were centered and scaled by *Z* score (Satija *et al.*, 2015). Following these filtering steps, 37 048 cells with 13 764 genes were left for downstream analysis. These cells were then visualized using UMAP (McInnes *et al.*, 2018) on 30 principal components and clustered with a resolution of 0.25 using the Louvain algorithm (Blondel *et al.*, 2008) as implemented in the Seurat framework, resulting in 9 clusters (Supplementary Fig. S1A) (Satija *et al.*, 2015). Another joint scRNA-seq and CITE-seq dataset of PBMCs with 228 surface protein antibodies from HIV vaccine trial patients in Hao *et al.* (2021) was processed in the same way as the aforementioned dataset. Following this processing, 56 775 cells with count data for 17 808 genes remained, separating into 19 clusters (Supplementary Fig. S1B).

### 2.2 ChIMP method

**Fig. 1.**
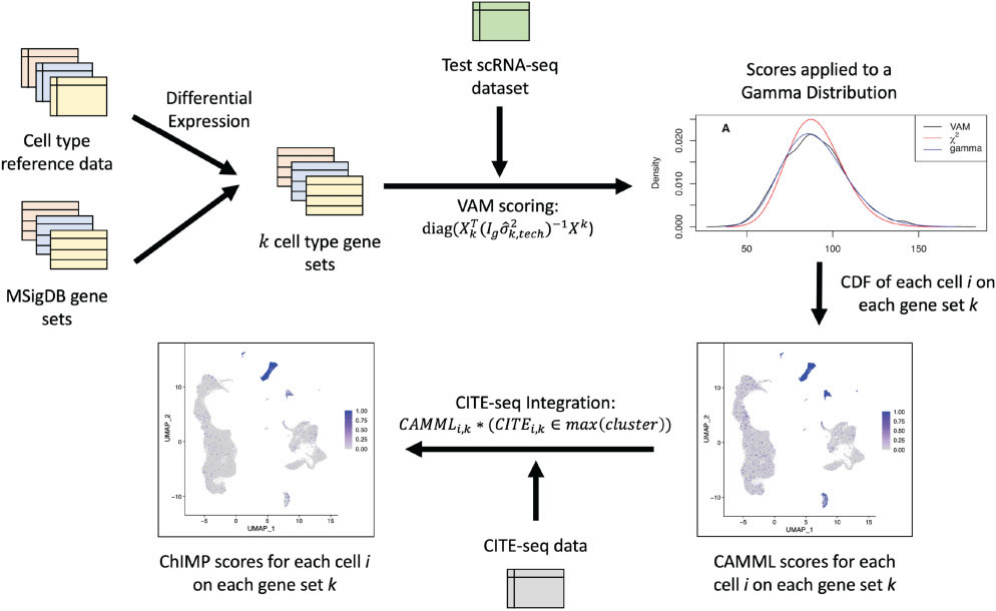
ChIMP pipeline. scRNA-seq data undergo VAM scoring based on reference cell type gene sets to receive CDF scores for cell types. These scores are then modified by the integration of CITE-seq data to produce ChIMP scores, visualized for B-cells on the Hao *et al.* (2021) data

**Fig. 2.**
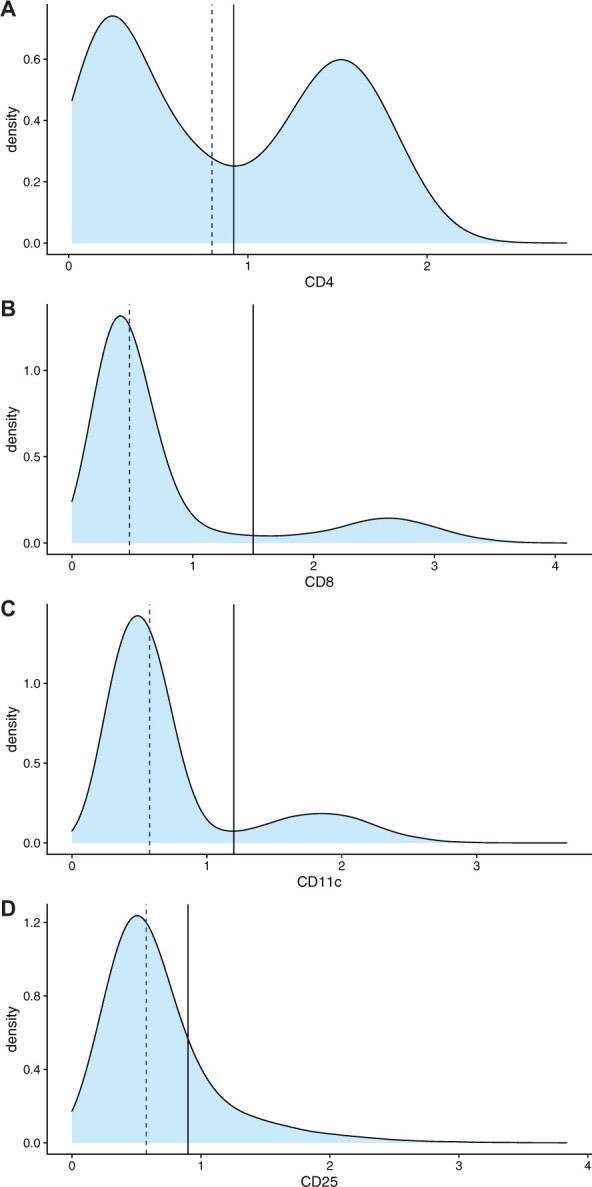
CITE-seq distributions. A visual of the densities of CITE-seq counts for four example markers (**A**) CD4, (**B**) CD8, (**C**) CD11c and (**D**) CD25 with the median marked with a dashed line and the k-means clustering cut-off marked with a solid line

To support cell typing of joint scRNA-seq/CITE-seq data, we have developed a simple but robust method for integrating CITE-seq data into the original CAMML method. The CAMML method is outlined in detail in the Supplementary Information but briefly: CAMML builds gene sets for cell types of interest by running DE analysis on reference data and intersecting that with existing cell type gene sets from the Molecular Signatures Database (MSigDB) (Schiebout and Frost, 2022). Cells are then scored with Variance Adjusted Mahalanobis (VAM) for the enrichment of the genes within a gene set, resulting in a cumulative distribution function (CDF) score between 0 and 1 (Frost, 2020). We have named the technique outlined in this article ‘CAMML with the Integration of Marker Proteins (ChIMP)’. As visualized in Figure 1, the ChIMP method performs multi-label cell typing of joint scRNA-seq/CITE-seq data using the following steps:

- For each cell type surface protein marker profiled via CITE-seq, k-means clustering of the CITE-seq count data with two centers is used as a method for binarization. In other words, if a marker’s count number in a given cell is in the lower count values cluster, the CITE-seq score becomes 0; if it is in the higher count values cluster, the CITE-seq score will be 1. In cases where more than one marker is sufficient for a cell type (i.e. CD4 and CD8 in T cells), if either marker is in the high value cluster, the score is assigned as 1. If neither marker is in the high value cluster, the score is 0. The discretization of k-means clustering was selected for its ability to robustly discern between the typically bimodal distributions of CITE-seq counts. When compared to another discretization option in the form of the median, the cut-off between k-means clusters proved to be more effective at discerning between the two peaks (Fig. 2). This is further more robust than median in that the divide does not require any given cell marker to be designated ‘present’ in half of cells but rather allows the number of cells that are positive for a marker to be variable, which is more consistent with the underlying biology (Dash *et al.*, 2011; Kleiveland, 2015).
- The discretized cell type CITE-seq scores for each cell are then multiplied by the associated CAMML scores, resulting in an overall cell type score of 0 if the CITE-seq count is in the lower count values cluster for a given marker, and maintaining the original CAMML score if the CITE-seq count is in the higher values cluster.

It is important to note that the technique used by ChIMP to integrate CITE-seq data can never increase the scores computed using just scRNA-seq data. This makes ChIMP a strictly conservative modification of the original CAMML method, i.e. it will only lower sensitivity and increase specificity for cell type classification. If the generated scores are transformed into *P*-values and used for inference, the ChIMP method will result in a more conservative test. There is further discussion on the options within ChIMP for user customization and statistical implications in the Supplementary Information.

### 2.3 Comparative cell typing methods

We performed a comparative evaluation of ChIMP against the original CAMML method, cell typing based on continuous CITE-seq values, cell typing based on discretized CITE-seq values, SingleR (Aran *et al.*, 2019), SCINA (Zhang *et al.*, 2019b) and Weighted Nearest Neighbor (WNN) (Hao *et al.*, 2021) cluster-based manual assignment and Seurat Reference Mapping (Hao *et al.*, 2021; Satija *et al.*, 2015). CAMML cell typing was done using gene sets built from genes with a logFC greater than 5 in edgeR (Robinson *et al.*, 2010) DE analysis of the Human Primary Cell Atlas (HPCA) (Aran *et al.*, 2019; Mabbott *et al.*, 2013). The DE genes were then intersected with the bone marrow cell type gene sets available in the C8 collection of the MSigDB, version 7.5.1 (Hay *et al.*, 2018; Liberzon *et al.*, 2011). These cell type gene sets were then scored with VAM, weighted by the logFC of each gene, resulting in cell-level scores for each supported cell type (Frost, 2020; Schiebout and Frost, 2022).

Continuous CITE-seq cell typing was performed to give each cell a score from 0 to 1 based on the abundance of surface protein markers for each cell type. This was accomplished by creating an empirical CDF (eCDF) for all the normalized and scaled CITE-seq counts for each cell surface marker (R Core Team, 2022). Individual cells were then scored based on the eCDF value associated with the cell-level marker abundance. This approach generates scores on the same scale as those generated by CAMML and ChIMP. In cases where a single-cell type label was needed, the cell type whose surface marker had the highest eCDF score was used. Throughout this manuscript, this method will be referred to as CITE-seq eCDF. Discretized CITE-seq cell typing was also used as a comparative approach given that discretization is utilized for the integration of CITE-seq data in ChIMP. For this approach, each cell is given a binary score for each cell type based on whether the CITE-seq count for a given surface marker belongs to the lower or higher value cluster of CITE-seq counts for that surface marker across all profiled cells.

SingleR (Aran *et al.*, 2019) was used as an independent comparative measure for cell typing accuracy. HPCA (Mabbott *et al.*, 2013) was used as the reference for this method using the following cell types: B cells, NK cells, T cells and monocytes. SCINA was also applied as a comparative measure for the same cell types (Zhang *et al.*, 2019b). SCINA runs on gene sets for cell typing, so the same genes used for CAMML and ChIMP were fed to SCINA, the contents of which are outlined in Supplementary Table S1 (Zhang *et al.*, 2019b). WNN (Hao *et al.*, 2021) cluster-based annotation was performed by combining the scRNA-seq and CITE-seq data using the WNN pipeline and manually assigning cluster identity based on differential expression of scRNA-seq and CITE-seq cell-type markers across clusters. Lastly, Seurat V4 Reference Mapping was also used for comparative evaluation (Hao *et al.*, 2021; Satija *et al.*, 2015). Cells assigned to the aforementioned cell types in Seurat’s reference were used to assign the cell types of the query dataset (Hao *et al.*, 2021; Satija *et al.*, 2015). The single-cell type labels called by SingleR, SCINA and Seurat were used for accuracy and cell proportion comparisons (Aran *et al.*, 2019; Hao *et al.*, 2021; Satija *et al.*, 2015; Zhang *et al.*, 2019b).

### 2.4 Differential expression

To better understand cases of discordant cell typing across CAMML and CITE-seq, VAM was performed on the Hao dataset (Hao *et al.*, 2021) for all Kyoto Encyclopedia of Genes and Genomes (KEGG) (Kanehisa and Goto, 2000) gene sets available in version 7.5.1 of MSigDB (Liberzon *et al.*, 2011). VAM CDF scores for each KEGG pathway were then compared by DE analysis between clusters via Wilcoxon Rank Sum test within Seurat’s ‘FindAllMarkers’ function (Satija *et al.*, 2015; Wilcoxon, 1945). The log fold change (logFC) threshold between groups was required to be greater than .01 and only upregulated pathways were considered. The same functions were also run on both the gene expression data and CITE-seq data with identical parameters.

### 2.5 Entropy analysis

To evaluate ChIMP’s specificity, we leveraged a method for measuring entropy previously applied in the CAMML manuscript: modified Shannon Diversity Index (mSDI), which is defined in the equation below (Schiebout and Frost, 2022). This allows cells to be scored based on both the strength of their ChIMP scores and the number of how many cells are present. In ordinary SDI, if two cells only had non-zero ChIMP scores for T cells (i.e. the scores for all other evaluated cell types are 0), but those T cell scores differed, they would still have identical entropy values. However, by modifying the SDI, the cell with a higher T cell score receives a lower score. The modification is a simple non-significant smoothing parameter (in this case set to 0.001) that adjusts ChIMP scores in order to eliminate scores of zero, allowing proportions to be considered with greater context.

$$\begin{array}{c} \text{mSDI}=-\sum_{i=1}^{R}p_{i}\text{ln}p_{i}\\ p_{i}=\frac{\text{ChIMP}+\epsilon }{\sum C\text{hIMP}}\\ i=\text{cell type score}\\ R=\text{number of scores in}\;a\;\text{cell} \end{array}$$

## 3 Results and discussion

### 3.1 Lawlor flow cytometry data

**Fig. 3.**
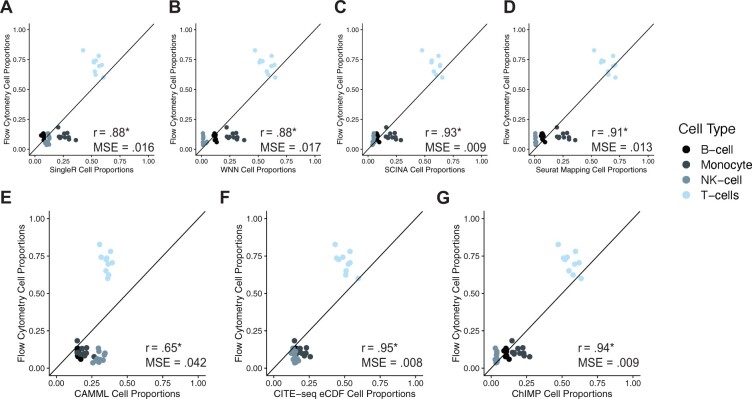
Flow proportions vs. SingleR, SCINA, WNN cluster annotation, Seurat Reference Mapping, CAMML, CITE-seq eCDF and ChIMP. *P<.001. The proportions of cell types identified in the joint scRNA-seq and CITE-seq samples from Lawlor *et al.* (2021) by (**A**) SingleR, (**B**) WNN, (**C**) SCINA, (**D**) Seurat Reference Mapping, (**E**) single-label CAMML, (**F**) CITE-seq eCDF and (**G**) ChIMP plotted against the proportions of cell types found in the flow cytometry samples. Each plotted pair of flow-based and scRNA-seq/CITE-seq-based cell proportions were computed using the same tissue sample that was divided prior to FACs and scRNA-seq/CITE-seq analysis. Pearson’s product moment correlation was used to determine the correlation and t-distribution based *P*-values

To compare ChIMP performance relative to CAMML, CITE-seq eCDF, SingleR, SCINA, WNN cluster annotation and Seurat Reference Mapping, we analyzed the joint scRNA-seq/CITE-seq and flow cytometry dataset compiled by Lawlor *et al.* (2021). To generate this dataset, flow cytometry and joint scRNA-seq/CITE-seq were performed in parallel on each experimental sample. While this approach does not provide ground truth cell type labels for the single-cell data, it allows the cell type proportions computed on the single-cell data to be compared to the proportions measured by flow cytometry. The original article performed flow sorting to manually discern four cell types: B cells, monocytes, NK cells and T cells. The proportions of each cell type called by SingleR, SCINA, WNN cluster annotation, Seurat Reference Mapping, single-label CAMML, single-label CITE-seq eCDF and single-label ChIMP were compared to these proportions for accuracy. In every case, the cell label proportions were positively correlated with the flow cytometry proportions, ranging from 0.65 to 0.95, and low mean squared error (MSE), ranging from 0.042 to 0.008 (Fig. 3). All tools were relatively efficient as well, with runtimes all less than 5 min. Of note, the inclusion of canonical markers, chosen for their status as markers of a given cell type (Zheng *et al.*, 2017) (as outlined in Supplementary Table S1), from CITE-seq data in ChIMP improves the correlation and MSE of the cell-typing proportions markedly compared to CAMML. The top cell type label based on the CITE-seq eCDF was the most correlated with the flow cytometry proportions, which is not surprising given that CITE-seq values are based on abundance of the same markers used for FAC sorting.

These results highlight two important considerations:

- It begs the question of the defining characteristics of a cell type. Given that cell sorting by surface markers has been a mainstay of cell biology research, its use for cell typing of single-cell data seems particularly vital. However, in cases where cell phenotype differs from cell surface protein markers, the exclusion of transcriptomic data may give an incomplete picture of cell state and function. While the CITE-seq eCDF method is the most consistent with previous bulk cell typing methods, the potential information gleaned from combining transcriptomic and surface protein abundance presents a novel direction for cell typing of single-cell data.
- It highlights the challenge of finding a gold standard method for benchmarking cell typing methods that is biased toward neither transcriptomic nor surface protein cell typing.

### 3.2 Entropy analysis

**Fig. 4.**
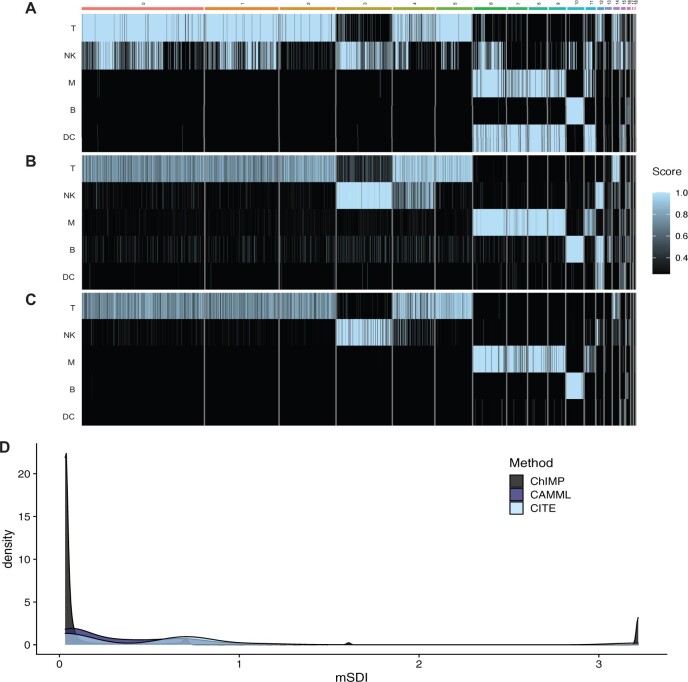
Entropy analysis of ChIMP on PBMCs. (**A–C**) Heatmaps of cell type scores (T, T cells; NK, NK cells; M, monocytes; B, B cells; DC, dendritic cells) for discretized CITE-seq, CAMML and ChIMP, respectively. (**D**) A density plot of the mSDI scores for each method on the Hao *et al*. (2021) joint scRNA-seq/CITE-seq data

Given the aforementioned difficulties with unbiased benchmarking of ChIMP, we decided to evaluate the entropy of cell types by computing the mSDI value for each cell in the Hao *et al.* (2021) joint scRNA-seq/CITE-seq dataset. Utilizing the mSDI gives a measure of each cell’s cell type entropy, with lower values representing cells that are confidently a single-cell type and higher values representing cells that are not confidently identified as any individual cell type. This serves as a useful measure to determine how differentiated a given cell is and how specific a cell typing method is. To evaluate whether cell type entropy was improved with ChIMP versus other multi-label cell typing approaches, we also computed the mSDI for cell types generated by discretized CITE-seq and CAMML. In this case, we performed cell typing for dendritic cells (DCs), B cells, monocytes, natural killer (NK) cells and T cells. The genes and CITE-seq markers used are outlined in Supplementary Table S1. Figure 4A–C shows the heatmaps of the scores for each method. This analysis revealed a marked reduction in the mSDI entropy measure when utilizing ChIMP (median mSDI of 0.047) relative to multi-label CITE-seq and CAMML (medians of 1.103 and 0.187, respectively) (Fig. 4D). ChIMP confidently assigned a single-cell type to each cell more often than either single-omics method (i.e, CITE-seq and CAMML).

These results illustrate the potential for ChIMP to serve as a conservative cell type identifier, especially when combined with the promising accuracy results outlined in Figure 3 and Supplementary Figures S4 and S5. Depending on the needs of a single-cell experiment, having a tool that integrates multiple modalities to make cell type predictions with high specificity could be useful for reducing the risk of type I error. Our entropy analysis of discretized CITE-seq, CAMML and ChIMP also highlights the notable discordance of cell type calls that can occur based on information from different modalities.

### 3.3 T cell/NK cell discordance

**Fig. 5.**
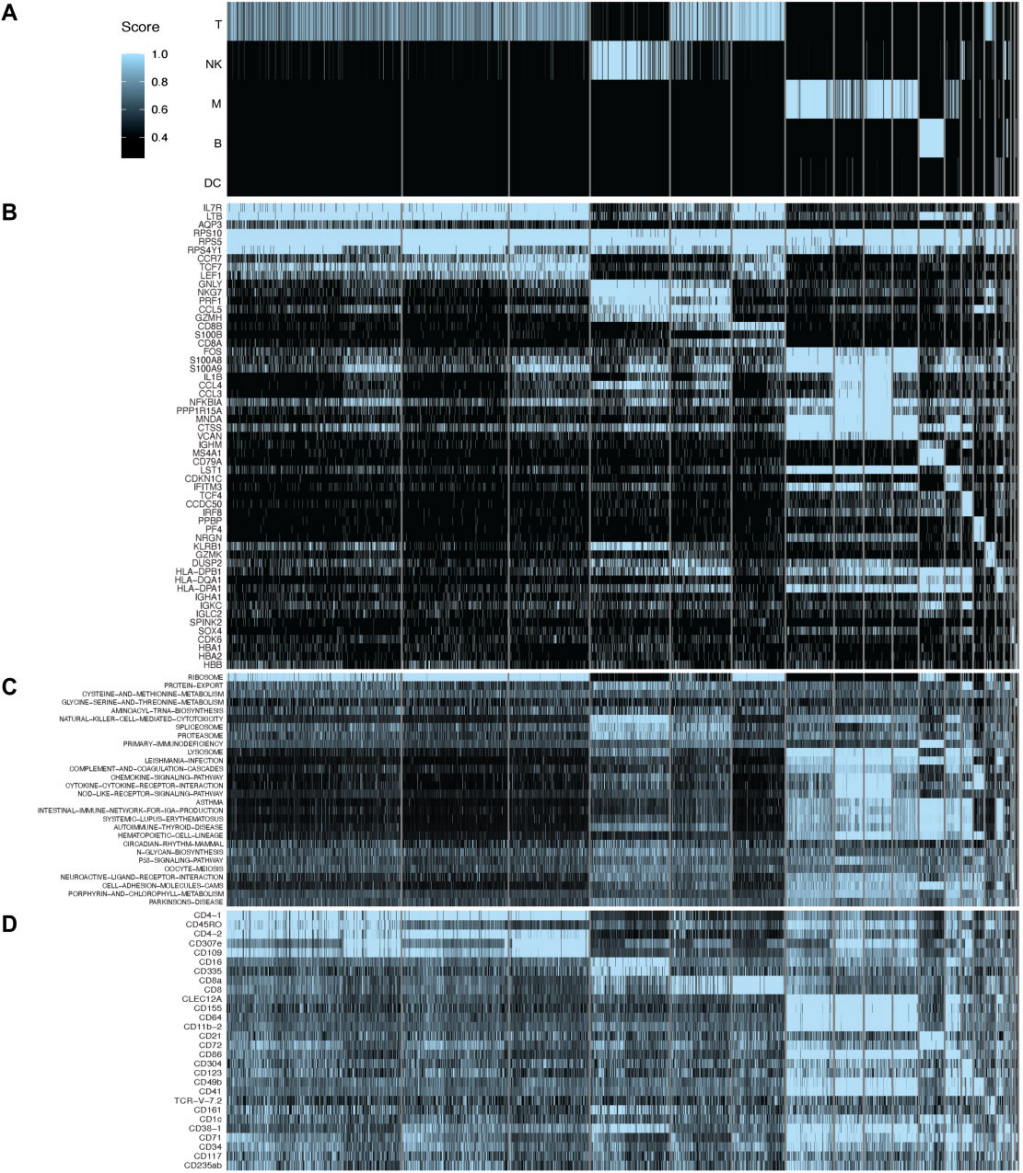
Discordant cell type classifications. Comparison of (**A**) the cell type scores for ChIMP, (**B**) the top DE genes across clusters, (**C**) the top DE KEGG pathways across clusters and (**D**) the top DE CITE-seq markers across clusters in the joint scRNA-seq/CITE-seq data from Hao *et al.* (2021)

The most common cell types found to have discordant classifications in our analysis were T cells and NK cells, especially cells with T cell-like CITE-seq signatures and NK cell-like transcriptomes, as seen in clusters 3 and 4 of the Hao *et al.* (2021)data. To investigate the nature of cells with this juxtaposition, VAM was used to generate cell-level scores for the KEGG (Kanehisa and Goto, 2000) pathways available in MSigDB (Liberzon *et al.*, 2011). These VAM-based pathway scores, along with the genes and CITE-seq markers, were analyzed for DE between clusters. The DE genes, VAM-scored KEGG gene sets and CITE-seq markers were sorted by logFC for each cluster with the top 3 visualized in a heatmap alongside the ChIMP cell type scores, as shown in Figure 5. We found that the DE pathways involved in cell cytotoxicity, such natural killer cell-mediated cytotoxicity, spliceosome and proteasome pathways, were highly significant (*P* < 0.001) for the discordant cell Clusters 3 and 4. This was further supported by the DE genes, where markers of cytotoxicity were also highly expressed in both Clusters 3 and 4. However, their CITE-seq markers were visibly distinct, with Cluster 3 expressing non-T cell markers: CD16 and CD335, and Cluster 4 expressing T-cell markers: CD8a and CD8.

Given that the transcriptomes of cells expressed markers of both T cells and NK cells (Fig. 4B), the inclusion of surface protein information is important to enable highly specific cell typing. In contrast, the use of surface protein markers alone without gene expression data limits the amount of information that can be gleaned regarding the phenotype of each cell. In this example, clarifying the cell types of discordant cells using CITE-seq gives no context to the highly cytotoxic phenotype occurring in some T cells, which may have a notable influence on the experimental model under investigation. By combining both CITE-seq and scRNA-seq information, ChIMP can consider the important phenotypic and surface protein information in conjunction. Further evaluation of discordant cells and ChIMP cell typing overall is available in the Supplementary Information, including ChIMP run on a dataset of joint scRNA-seq/CITE-seq from Mucosa Associated Lymphoid Tissue (MALT) tumor tissue (10k Cells from a MALT Tumor, 2018) and ChIMP utilized to discern T cell subtypes in the Hao *et al.* (2021) PBMC data.

## 4 Conclusion

The advent of single-cell transcriptomics has created unique opportunities for tissue characterization, particularly in diseased states (Azizi *et al.*, 2018; Davidson *et al.*, 2020; Qiu *et al.*, 2018; Wagner *et al.*, 2019). However, analysis of this new single-cell data is limited by increased noise and significant sparsity. Investigating the cell types present in these datasets is an ongoing challenge that requires compensating for these data quality challenges (Haque *et al.*, 2017; Kolodziejczyk *et al.*, 2015). To accomplish this while also addressing the tendency for cell types to occur on a continuum, we developed CAMML, a gene set-based multi-label cell typing tool for scRNA-seq data (Schiebout and Frost, 2022). CAMML performs well compared to existing single-label methods and has the added benefit of identifying cells that do not differentiate well into a single category, making it flexible for the analysis of intermediate states and stemness (Schiebout and Frost, 2022).

With the increasing utilization of multi-omic single-cell assays, we extended CAMML to support cell typing of joint scRNA-seq/CITE-seq data to more confidently and conservatively identify cell types. This new technique, ChIMP, performs multi-label cell typing that accounts for both the expression of cell type transcriptional signatures and abundance of cell type surface protein markers. When comparing ChIMP to ground truth methods of cell typing, ChIMP performs better or comparably with existing cell-level methods for cell type identification, both in accuracy and time efficiency. Furthermore, ChIMP successfully eliminates false positives that occur when each omics modality is used in insolation, resulting in a lower entropy score while maintaining multiple cell type labels when supported by both modalities. ChIMP is a conservative method for multi-label cell typing of joint scRNA-seq/CITE-seq data based on a statistical model that is robust, transparent and easily customizable.

## Supplementary Material

btac674_Supplementary_Data

## Data Availability

All datasets used in this article are publicly and freely available. The datasets were derived from sources in the public domain: Lawlor, et al. (https://data.humancellatlas.org/explore/projects/efea6426-510a-4b60-9a19-277e52bfa815), Hao, et al. (https://www.ncbi.nlm.nih.gov/geo/query/acc.cgi?acc=GSE164378), and MALT (https://www.10xgenomics.com/resources/datasets/10-k-cells-from-a-malt-tumor-gene-expression-and-cell-surface-protein-3-standard-3-0-0).
