# Supplementary material for: CAMML with the Integration of Marker Proteins (ChIMP)
Source: Bioinformatics. 2022 Oct 10;38(23):5206–13. doi: 10.1093/bioinformatics/btac674 (PMC9710548; doi:10.1093/bioinformatics/btac674)
Supplement: btac674_Supplementary_Data [file btac674_supplementary_data.docx]

**Supplementary Information**

**Supplementary Methods**

1. **CAMML method**

To ameliorate the notable challenges of noise, sparsity, and continuous phenotypes for cell typing scRNA-seq data, we previously developed cell typing using variance Adjusted Mahalanobis distances with Multi-Labeling (CAMML) [(Schiebout and Frost, 2022)](https://www.zotero.org/google-docs/?lM6bNU). CAMML leverages cell type gene sets through Variance-Adjusted Mahalanobis (VAM) [(Frost, 2020)](https://www.zotero.org/google-docs/?Dc3eFL) distance scoring to provide single or multi-label cell typing of scRNA-seq data. The Variance-Adjusted Mahalanobis (VAM) method computes cell-level gene set scores using the squared value of a modified Mahalanobis multivariate distance that is measured from the origin rather than the multivariate mean and adjusts for only the technical variance of the genes in each set rather than the full sample covariance matrix [(Frost, 2020)](https://www.zotero.org/google-docs/?xHRFl0). These squared modified Mahalanobis distances are computed on both the original scRNA-seq data and data where the cell labels are permuted to break inter-gene correlation. To determine a null distribution for these distances, a gamma distribution is fit to the non-zero distances for the permuted data. Scores for each cell are computed using this null distribution by finding the cumulative distribution function (CDF) value for the non-permuted squared distances resulting in a score from 0 to 1 for each cell. These scores are robust to large values, can be compared between gene sets of different sizes and can be subtracted from 1 to generate valid p-values under the null of uncorrelated technical noise [(Frost, 2020)](https://www.zotero.org/google-docs/?w6csRM). To allow for prioritization of particular genes in a given set, we updated VAM to accept positive gene weights [(Frost, 2020; Schiebout and Frost, 2022)](https://www.zotero.org/google-docs/?SCQklA). These gene weights are used to modify the technical variance for each gene by dividing the original technical variance estimate by the associated gene weight [(Schiebout and Frost, 2022)](https://www.zotero.org/google-docs/?C0vlQB). This adjustment prioritizes genes with large weights by reducing the associated variance penalty in the modified Mahalanobis distance computation. For the CAMML method, gene-level weights are used to prioritize genes that are highly characteristic of a cell type (i.e. CD8a in CD8+ T-cells) in the computation of VAM scores for cell type gene sets [(Frost, 2020; Schiebout and Frost, 2022)](https://www.zotero.org/google-docs/?tNaSfs). Support for gene weights is included in the 1.0.0 version of the VAM R package, which is available on CRAN.

Building on the weighted version of VAM, we developed the CAMML method and associated R package for multi-label cell typing of scRNA-seq data [(Schiebout and Frost, 2022)](https://www.zotero.org/google-docs/?2b5oiA). CAMML uses the weighted version of VAM to compute cell-level scores for gene sets representing cell types. These cell type-specific gene sets are defined to include both genes differentially expressed (DE) in the cell type as well as genes annotated to one of the MSigDB C8 [(Liberzon *et al.*, 2011)](https://www.zotero.org/google-docs/?RoV8je) gene sets associated with that cell type. The inclusion criteria and associated weight for DE genes is based on either the log-fold change in expression in the target cell type relative to other cell types or the -log(p-value) from the edgeR [(Robinson *et al.*, 2010)](https://www.zotero.org/google-docs/?LWz007) DE test. These genes are then intersected with the associated MSigDB C8 cell-type collection to designate the final gene set. These cell type gene sets can then be scored for a query scRNA-seq dataset using the weighted VAM algorithm outlined above and these scores can be leveraged to classify cells as desired [(Frost, 2020; Schiebout and Frost, 2022)](https://www.zotero.org/google-docs/?CYFhRg). The gene sets used in the analyses outlined in this paper are detailed in Supplementary Table 1.

1. **MALT data processing and filtering**

The final dataset utilized for evaluation was joint scRNA-seq/CITE-seq of Mucosa-Associated Lymphoid Tissue (MALT) B cell tumors [(10k Cells from a MALT Tumor, 2018)](https://www.zotero.org/google-docs/?6IjXl4). Because this dataset only profiled about 9500 cells, a less stringent Seurat filtering pipeline was applied to maintain cell numbers [(Satija *et al.*, 2015)](https://www.zotero.org/google-docs/?Zkjb0x). Specifically, only cells with at least 100 genes with nonzero counts, of which no more than 10% were mitochondrial, were kept for analysis, and any genes present in fewer than 10 cells were removed. These cells were then scaled and normalized in the same way as the other two datasets, but were visualized using UMAP [(McInnes *et al.*, 2018)](https://www.zotero.org/google-docs/?k4utcj) on only 10 principal components and clustered with the Louvain algorithm [(Blondel *et al.*, 2008)](https://www.zotero.org/google-docs/?DdUKUv) in Seurat with a resolution of .5 [(Satija *et al.*, 2015)](https://www.zotero.org/google-docs/?sBAECU). Following this, the dataset contained 6,438 cells in 11 clusters (Supplementary Figure 1C).


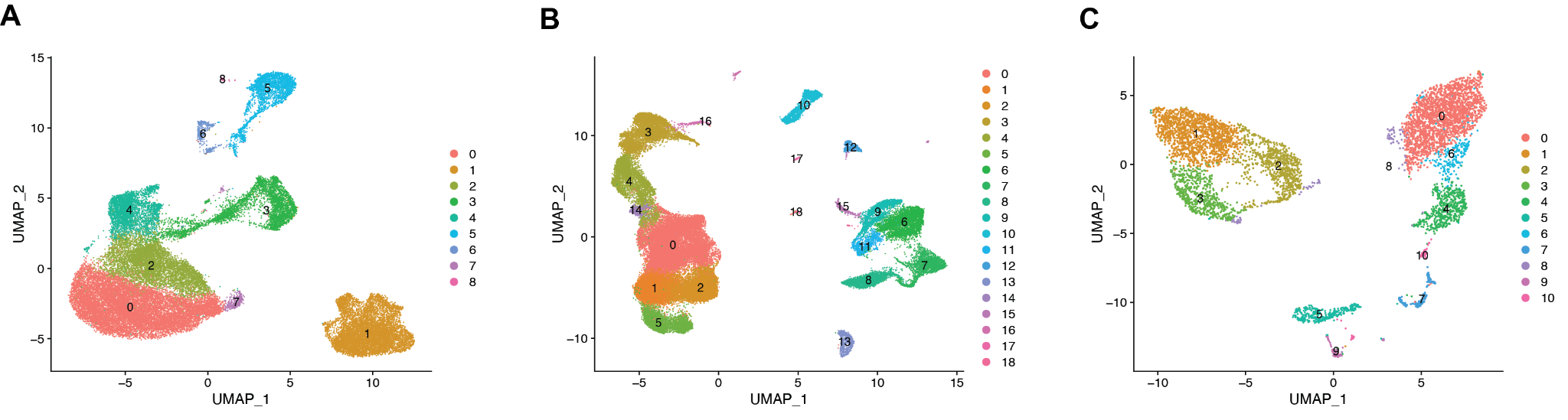


**Supplementary Figure 1. Visualization of the UMAPs for each of the joint scRNA-seq/CITE-seq datasets. A** and **B** were performed on 30 principal components with a cluster resolution of .25. **C** was performed on 10 principal components with a cluster resolution of .5. All visualizations were accomplished with Seurat [(Satija *et al.*, 2015)](https://www.zotero.org/google-docs/?f1VHNW).

1. **Accuracy analysis and subtyping**

In order to gain a clearer picture of the performance of ChIMP, we performed comparative analyses of SCINA, SingleR, and ChIMP on the manual cell type annotations provided in the Hao. et al. PBMC dataset [(Zhang *et al.*, 2019; Aran *et al.*, 2019; Hao *et al.*, 2021)](https://www.zotero.org/google-docs/?Qll0wL). While these annotations are not necessarily a ground truth, comparing ChIMP’s results to them gives an estimate of its consistency with other methods and an additional measure of performance. To compare ChIMP’s results from analysis of the Hao, et al. data outlined in Figure 3, an additional layer of T cell subtyping was performed to match the existing annotations. The T cell subtyping was accomplished by first isolating all cells identified as T cells in the ChIMP cell type analysis previously performed on this data. These cells were then reanalyzed with ChIMP on gene sets distinct to T cell subtypes for CD4+ and CD8+ T cells, as determined by differential expression analysis of HPCA data [(Mabbott *et al.*, 2013; Aran *et al.*, 2019; Robinson *et al.*, 2010)](https://www.zotero.org/google-docs/?bZbQ0C). Of note, due to the high transcriptional similarity between T cell subtypes, the logFC cut-off was lowered to 2 in order to have satisfactorily comprehensive gene sets for analysis. Any cells that were identified as unknown by ChIMP were labeled “other” in the first round of analysis and “other T” in the T cell specific analysis so as to be consistent with the labeling convention used in the cell annotations provided by Hao, et al [(Hao *et al.*, 2021)](https://www.zotero.org/google-docs/?6uslj8). A similar process was performed for both SCINA and SingleR, whereby the major cell types were identified, followed by a T cell subtyping step [(Aran *et al.*, 2019; Zhang *et al.*, 2019)](https://www.zotero.org/google-docs/?wH76jW). As outlined previously, the same gene sets used by ChIMP were provided for SCINA. Following this, the major cell types annotated by Hao, et al. were compared to those identified by ChIMP, SCINA, and SingleR. Pearson’s product-moment correlation was performed to compare the cell proportions of each method compared to the proportions of annotated cell types in Hao, et al [(Hao *et al.*, 2021; R Core Team)](https://www.zotero.org/google-docs/?3V5P5U). Additionally, accuracy, in the form of the percent of all identifications that were correct identifications according to the cell annotations, and Pearson’s chi-square statistics were performed to further understand the performance of each method. Given that SingleR does not provide an “other” designation for cell types, its chi-square test was unsuccessful [(Aran *et al.*, 2019)](https://www.zotero.org/google-docs/?tgW6nn). An additional test was done with zero-sum categories removed, but it should be noted this statistic is not directly comparable to the chi-square statistics of the other methods due to its differing input.

In addition to this analysis on the major cell types, subtyping for more granular T cell populations was also carried out. The method for accomplishing this follows a very similar process to the one above. For ChIMP, T cells were isolated and subtyping was performed based on gene sets built from differential expression analysis [(Mabbott *et al.*, 2013; Aran *et al.*, 2019; Robinson *et al.*, 2010)](https://www.zotero.org/google-docs/?BDHcUy). The T cell subtypes selected for analysis were as follows: naive CD8+ T cells, CD8+ memory T cells, naive CD4+ T cells, regulatory T cells, CD4+ memory T cells, and miscellaneous other T cells. The CITE-seq surface markers chosen for cell typing by ChIMP were selected for their canonical association with the aforementioned cell types [(Zheng *et al.*, 2017; De Biasi *et al.*, 2020)](https://www.zotero.org/google-docs/?sj97kz). SCINA and SingleR were utilized in a similar fashion, whereby T cells were isolated and then classified into one of the aforementioned T cell subtypes [(Aran *et al.*, 2019; Zhang *et al.*, 2019)](https://www.zotero.org/google-docs/?I1ACKD). Again, the T cell subtype gene sets used by ChIMP were provided for SCINA. The results from each method were then compared to the Hao, et al. annotations and evaluated with the same statistics mentioned above [(Hao *et al.*, 2021)](https://www.zotero.org/google-docs/?BNMWiB). The genes and surface markers used in these analyses can be viewed in Supplementary Table 2.

**Supplementary Results and Discussion**

1. **MALT scRNA-seq/CITE-seq data**

To validate this metric on another dataset and in a different experimental condition, the 10x Genomics joint scRNA-seq/CITE-seq dataset of MALT B cell tumors [(10k Cells from a MALT Tumor, 2018)](https://www.zotero.org/google-docs/?zgYhSV) was scored for discretized CITE-seq, CAMML, and ChIMP, and mSDI was evaluated in each case (Supp. Figure 2). We found that, not only did ChIMP again have the lowest median mSDI (0.068 versus 0.622 for CAMML and 0.701 for CITE-seq), but also that both CAMML and CITE-seq had false positive cell type scores that were ameliorated by the integration of both methods in ChIMP. Specifically, the NK cell marker in the CITE-seq data scores highly in almost every cluster, but there is little indication of a strong NK cell signal with the transcriptomic data, so the signal is removed in ChIMP. Conversely, CAMML scores highly for macrophages in almost every cluster, and while there is some indication of a macrophage population present in clusters 1-3 and 9, the inclusion of CITE-seq removes what is likely to be a false signal in clusters 0 and 4. This false transcriptomic signal is likely due to B cells expressing a gene or pathway that is also associated with macrophages. Indeed, in Supp. Figure 3, which shows the top differentially expressed genes, KEGG pathways, and CITE-seq markers in the MALT B cell tumor data, there are genes and pathways that are upregulated in both the B cell and macrophage clusters. This data also illustrates ChIMP’s ability to maintain multiple cell type labels when they are supported by both modalities, as can be visualized with T cells and macrophages in clusters 2-4. It is likely that these clusters either have mixed populations of singleton cells or interacting doublets and ChIMP is still able to identify the potential for multiple cell types in this case–a trait of the original CAMML method that was important to maintain—even with a more conservative method.

1. **Accuracy analysis of major cell types**

A comparison of the performance of SCINA, SingleR, and ChIMP was performed on the Hao, et al. dataset major cell type annotations in order to provide an additional comparative evaluation of the performance of ChIMP [(Zhang *et al.*, 2019; Aran *et al.*, 2019; Hao *et al.*, 2021)](https://www.zotero.org/google-docs/?gvntTX). As can be visualized in Supp. Figure 4, of the three methods, ChIMP had the highest accuracy compared to the manual cell type annotations at 88.5% (versus SingleR at 86.7% and SCINA at 43.2%). ChIMP and SingleR also performed comparably when evaluating the Pearson correlation of their cell type proportions relative to those annotated by Hao, et al [(Hao *et al.*, 2021)](https://www.zotero.org/google-docs/?vcPZQK). While this is not necessarily a ground truth accuracy evaluation, the high consistency between the dataset’s original annotations and ChIMP’s identifications illustrates promise for ChIMP’s consistency with what might be expected of a dataset. Furthermore, ChIMP is designed to be a highly specific method, favoring non-identification over false identification, resulting in a high volume of “other” cells. While this certainly affects the accuracy, it allows for greater user confidence for high-scoring cell types.

1. **T cell subtyping**

In order to test ChIMP on more highly specific cell subtypes, we performed cell typing with ChIMP, SCINA, and SingleR on T cell subtypes in the Hao, et al. PBMC data [(Hao *et al.*, 2021)](https://www.zotero.org/google-docs/?4eL47N). ChIMP again performed most accurately relative to the annotations available within the dataset, with an accuracy of 64.6%, versus 61.1% and 49.2% for SingleR and SCINA respectively (Supp. Figure 5) [(Zhang *et al.*, 2019; Aran *et al.*, 2019)](https://www.zotero.org/google-docs/?h7isUL). Of note, SingleR has the best correlation of cell type proportions relative to the annotated cell types, but this is likely a result of a high volume of zeros and an imbalanced dataset. ChIMP again performed with intentionally high stringency, resulting in many cells falling in the “other” category. In fact, when cells identified by ChIMP as “other” are excluded from the accuracy calculation, ChIMP’s performance increases to 80%, indicating high confidence in cell subtype identification, especially among subtypes that often have notable overlap in expression and markers.

1. **Customization and inference**

ChIMP allows for the integration of scRNA-seq and CITE-seq to be easily and efficiently customized. The genes used for gene set scoring can be designated and weighted according to an investigator's needs, either by completely customizing the gene set information or setting the desired differential expression logFC cut-off and gene weights within the CAMML Comprehensive R Archive Network (CRAN) package [(Schiebout and Frost, 2022; R Core Team)](https://www.zotero.org/google-docs/?58cJUD). The analyses outlined here rely on Signature Gene Set in MSigDB, which can be a limitation, both in its reliance on the generalizability of this data and in its limited number of cell types [(Liberzon *et al.*, 2011)](https://www.zotero.org/google-docs/?Ah700S). However, users can also customize their own cells to integrate genes and gene weights independently important to their research.

Additionally, the markers used for CITE-seq discretization can be customized, both by designating which markers are evaluated for each cell type and by customizing the nature of the required markers. For example, if numerous markers are used for a single cell type, each marker can be required to be in the higher cluster for the CITE-seq score to be 1, or the score can be set to 1 as long as one of the markers is in the higher cluster. Of note, while more than one marker can be used for specificity, ChIMP does not require an abundance of marker data for functionality. For granular cell types, ChIMP only requires a single marker per cell type to successfully integrate CITE-seq information, though more markers can be used to increase specificity, although a potential loss of sensitivity may occur. Additionally, further customizability was added to ChIMP in the form of selecting for the absence of a marker (i.e. if an investigator is attempting to identify non-immune cells, the lower cluster for CD45 can be positively selected for instead of the higher cluster). Furthermore, a quantile cut-off of any value for marker counts can be used rather than a k-means clustering discretization in cases where a user wants to manually increase or decrease the influence CITE-seq data has on the cell type scores. This allows users to integrate cases where surface markers of specific concentrations beyond present or absent are necessary for cell type identification, particularly in cell subtyping. Lastly, while not illustrated in the analyses performed here, ChIMP can be utilized on scRNA-seq/CITE-seq data from mice in addition to humans.

The previously developed CAMML method allows for statistical inference regarding cell type identity via the generated CDF scores [(Schiebout and Frost, 2022; Frost, 2020)](https://www.zotero.org/google-docs/?2WoOXk). While the ChIMP method alters these scores, multiple hypothesis correction and inference can still be used. Specifically, ChIMP provides more conservative control of the type I error rate than CAMML, i.e., the CAMML-generated p-values are unaltered when the discretized CITE-seq score is 1 or are set to 1 when the CITE-seq score is 0. This correction does not change the inferential capabilities of the scores, but rather makes the evaluation more stringent, i.e., ChIMP reduces the type I error rate at the expense of lower power.


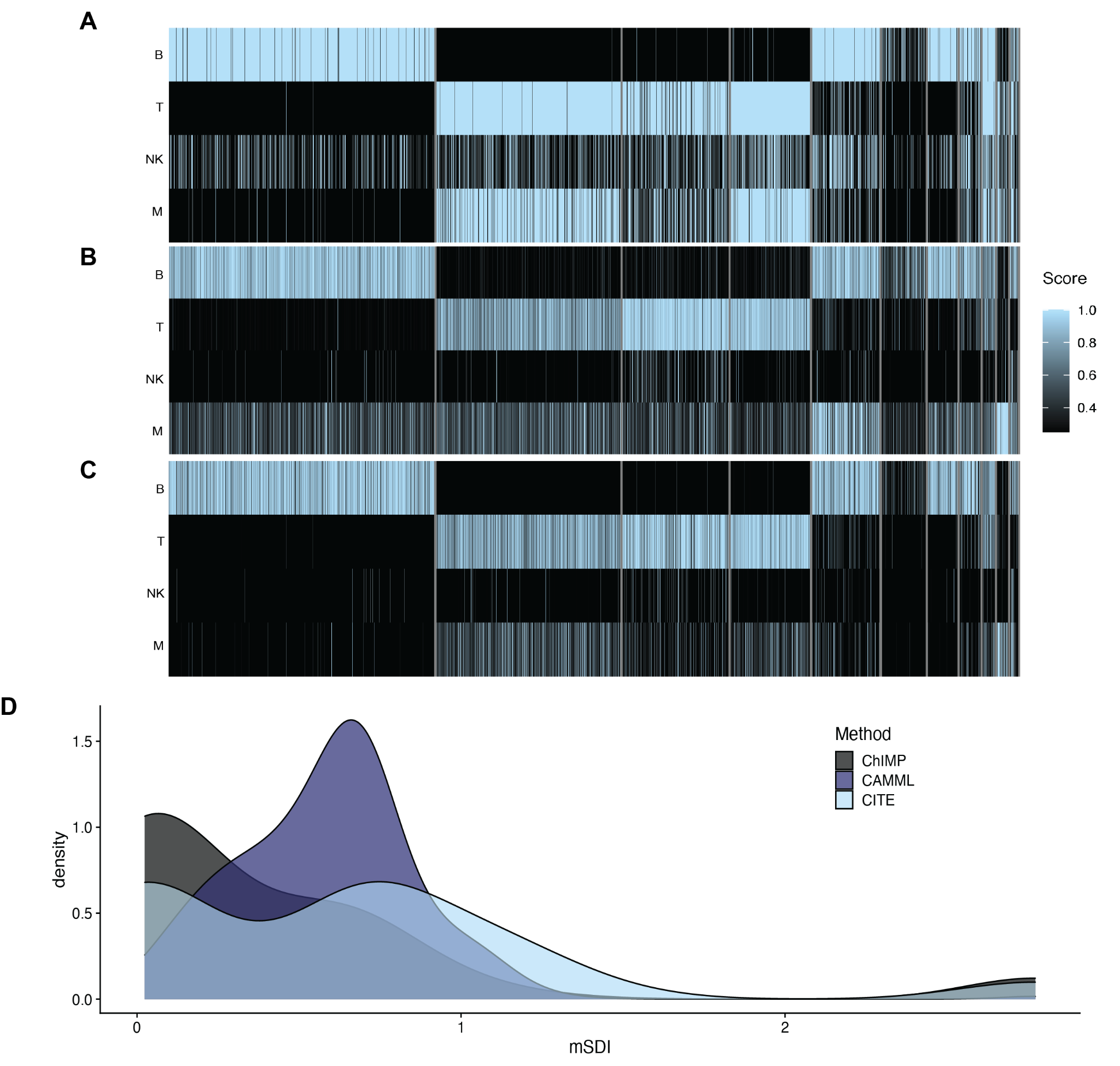


**Supplementary Figure 2. Entropy analysis of ChIMP on MALT B cell tumor data. A-C.** Heatmaps of cell type scores (B: B cells, T: T cells, M: macrophages, NK: NK cells) for discretized CITE-seq, CAMML, and ChIMP respectively. **D.** A density plot of the mSDI scores for each method on the MALT joint scRNA-seq/CITE-seq data.[(Li *et al.*, 2021)](https://www.zotero.org/google-docs/?zwlncm)

**
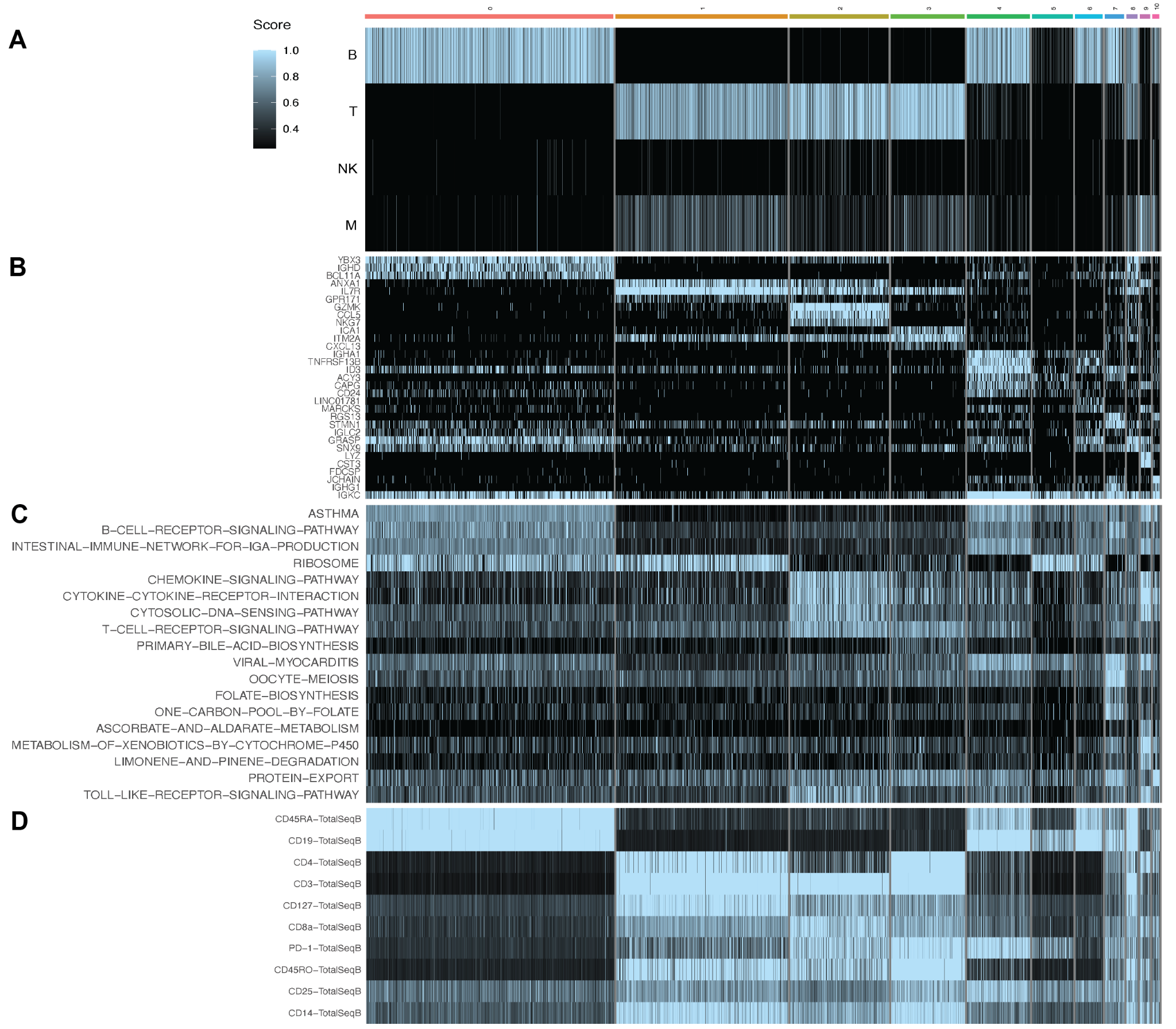
**

**Supplementary Figure 3. Differential Expression Analysis in MALT B cell tumor data** [(10k Cells from a MALT Tumor, 2018)](https://www.zotero.org/google-docs/?mcMQNk). Comparison of **A.** the cell type scores for ChIMP, **B.** the top differentially expressed genes across clusters, **C.** the top differentially expressed KEGG pathways across clusters, and **D.** the top differentially expressed CITE-seq markers across clusters in the joint scRNA-seq/CITE-seq data from 10x Genomics [(10k Cells from a MALT Tumor, 2018)](https://www.zotero.org/google-docs/?TLqYSZ).


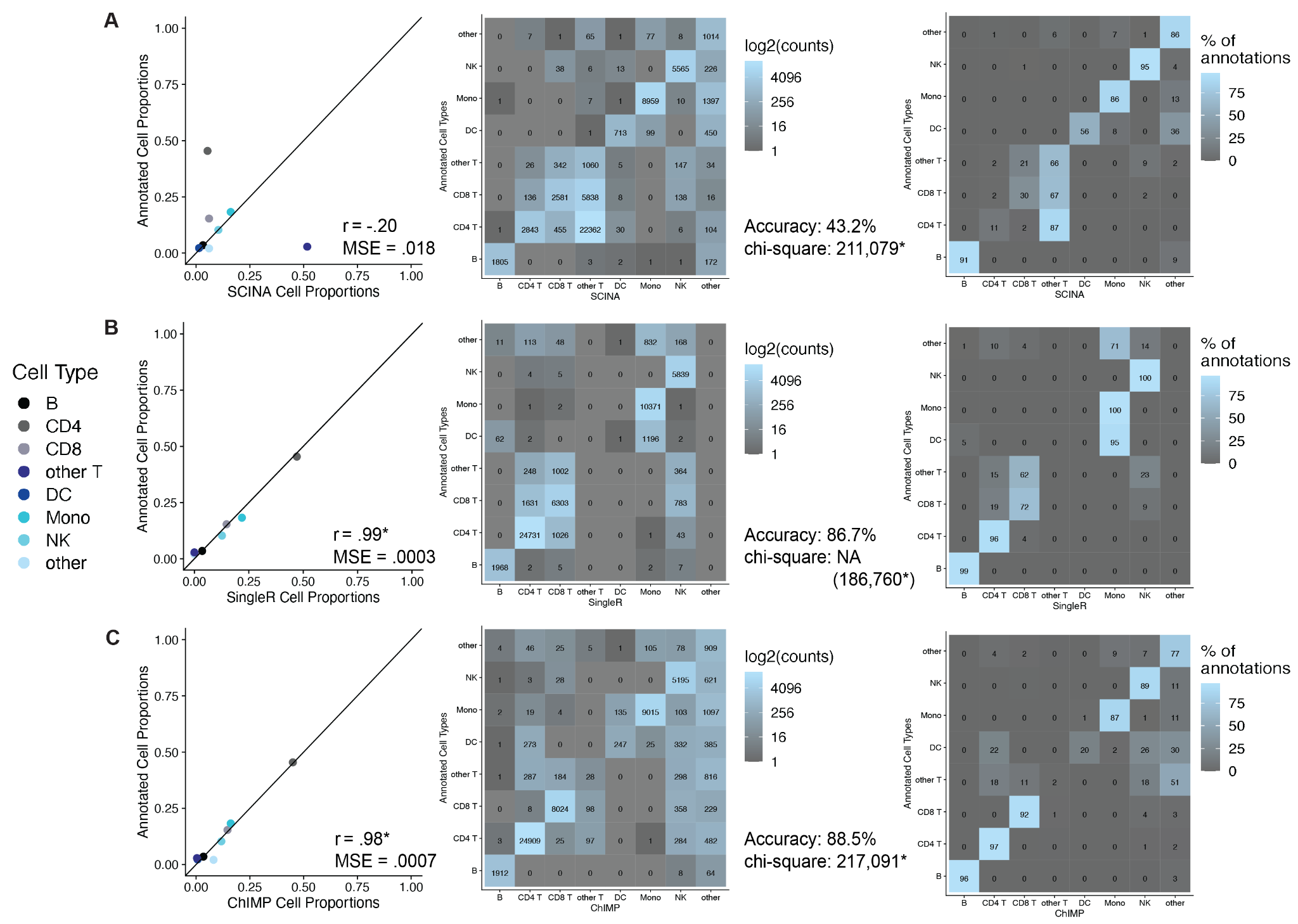


**Supplementary Figure 4.** **Proportions and confusion matrices of identified cell types with SCINA, SingleR, and ChIMP on the Hao, et al dataset** [(Hao *et al.*, 2021)](https://www.zotero.org/google-docs/?4A5SEd). Proportions of each cell type as annotated by Hao, et al. versus the proportions identified by each method and confusion matrices of the same comparison in counts and percents for **A.** SCINA, **B.** SingleR, and **C.** ChIMP. Of note, Pearson’s chi-square test is not successful on SingleR due to its zero-sum columns. A Pearson’s chi-square statistic with those columns removed is provided in parenthesis but is not fully comparable to the other methods’ chi-square statistics due to its different dimensions.


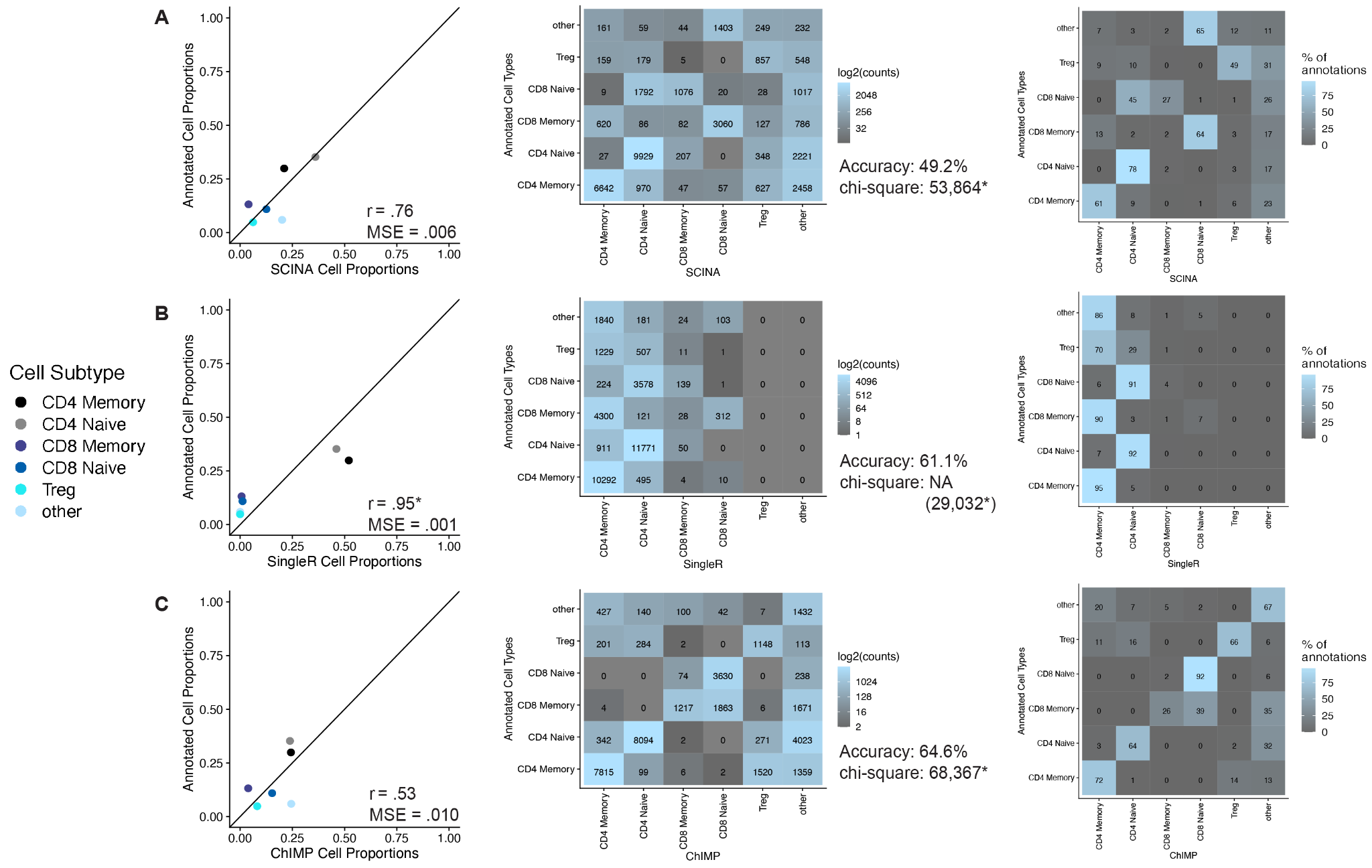


**Supplementary Figure 5.** **Proportions and confusion matrices of T cell subtyping with SCINA, SingleR, and ChIMP.** Proportions of T cell subtypes from Hao, et al. [(Hao *et al.*, 2021)](https://www.zotero.org/google-docs/?mA31JT) versus the proportions identified by each method and confusion matrices of the same comparison by count and percent for **A.** SCINA, **B.** SingleR, and **C.** ChIMP. Of note, Pearson’s chi-square test is not successful on SingleR due to it not identifying any T cell as regulatory T cells and its lack of a designation for “other” cells. An additional Pearson’s chi-square statistic with those empty columns removed is provided in parenthesis but, of note, this statistic is not comparable to the other chi-square statistics due to its altered dimensions.

| Cell Type | scRNA-seq Genes | CITE-seq Markers |
| --- | --- | --- |
| B cells | AIM2 (5.52), BANK1 (8.27), BLK (7.66), BTNL9 (6.42), CD19 (8.89), CD22 (6.05), CD79A (8.70), CPNE5 (6.85), E2F5 (7.28), FCRL1 (8.56), FCRL2 (6.64), FCRL3 (5.17), FCRLA (8.64), HLA-DOB (6.90), LINC00926 (5.38), MS4A1 (8.07), P2RX5 (6.50), PKHD1L1 (6.07), PLEKHG1 (5.29), PNOC (7.78), RALGPS2 (5.14), SPIB (6.32), STAP1 (5.66), TLR10 (7.26) | CD19 |
| T cells | BCL11B (6.20), CD2 (5.47), CD3D (7.28), CD3E (5.64), CD3G (8.35), CD8A (6.41), CD8B (8.00), GZMK (5.49), ICOS (6.36), INPP4B (6.34), ITK (5.86), ITM2A (5.35), KLRG1 (5.65), LEF1 (5.72), LRRN3 (7.70), MAL (7.54), NELL2 (7.07), OXNAD1 (5.16), RNF157 (5.55), SH2D1A (5.06), SIRPG (5.73), TC2N (5.69), TCF7 (6.38), THEMIS (7.43), TRAC (5.82), TRAT1 (7.90), TRBC1 (5.39), UBASH3A (5.51) | CD8A or CD4 |
| Natural killer cells | CLIC3 (7.45), FASLG (5.98), GZMB (5.33), IL18RAP (5.63), KIR2DL1 (5.02), KIR2DL4 (5.88), KIR3DL1 (6.62), KIR3DL2 (5.86), KLRF1 (6.05), KRT86 (5.44), PRR5L (5.60), PTGDR (5.34), S1PR5 (5.35), SH2D1B (7.83), SLFN13 (5.57), XCL1 (6.65), YES1 (5.55) | CD56 |
| Monocytes | FCN1 (7.36), FPR1 (5.06), RBP7 (6.50), RNASE2 (5.36), S100A12 (5.22), TMEM170B (5.50), VCAN (6.70) | CD14 |
| Dendritic cells  (used in the Hao, et al. dataset cell-typing) | CD1E (7.19), CLIC2 (5.60), DNASE1L3 (6.90), MRC1 (5.97) | CD11C |
| Macrophages  (used in the MALT dataset cell-typing instead of Monocytes) | ADGRE2 (7.68), C1QA (10.21), C1QB (10.86), C5AR1 (8.96), CD300LF (6.35), CD68 (7.64), CSF1R (6.04), CTSL (6.07), CXCL16 (7.74), FGR (5.06), FMNL2 (6.14), FTH1 (5.10), HCK (6.74), HK3 (7.30), ITGAX (5.68), LILRA2 (5.64), LILRB2 (6.24), LINC01503 (5.28), MRAS (5.59), MS4A7 (6.88), MSR1 (6.77), PAPSS2 (5.84), PILRA (7.18), SLC31A2 (6.20), SLC7A7 (5.59), SMPDL3A (5.12), SOD2 (5.84), TBC1D8 (5.02), VMO1 (6.20) | CD14 |

**Supplementary Table 1. The gene sets and CITE-seq markers used for cell-typing.** For each gene, the value following it in parentheses is its gene weight. As noted in the table, the dendritic cell and macrophage gene sets are used only in specific datasets. The CITE-seq markers selected for these analyses reflect canonical markers of each cell type [(Zheng *et al.*, 2017)](https://www.zotero.org/google-docs/?7DItv3).

| Cell Type | scRNA-seq Genes | CITE-seq Markers |
| --- | --- | --- |
| CD4+ T cells | ANKRD55 (5.31), CD4 (5.1), ADTRP (4.44), ST6GALNAC1 (4.31), RTKN2 (4.28), FHIT (4.16), FCER1A (4), TMEM176B (3.98), CD40LG (3.65), CYSLTR1 (3.62), IGSF6 (3.24), TRAV23DV6 (3.19), DACT1 (3.18), LGALS2 (2.98), SLC40A1 (2.93), RNF175 (2.9), CMTM8 (2.88), ZC3H12D (2.86), LY96 (2.76), ARL17B (2.72), IL6R (2.71), CSTA (2.68), TNFRSF4 (2.67), CSGALNACT1 (2.65), ST8SIA1 (2.64), F5 (2.62), MS4A6A (2.6), CPVL (2.6), TIAM1 (2.6), CSF2RB (2.58), XIST (2.53), ANKRD36BP2 (2.52), BAG3 (2.46), CCR6 (2.44), BIRC3 (2.41), IFI30 (2.38), KISS1R (2.37), FCER1G (2.33), TRAV9-2 (2.31), FRMD4B (2.3), AP3M2 (2.29), CLEC4A (2.28), LIMS1 (2.27), LPAR6 (2.27), SERPINA1 (2.24), ICOS (2.23), CPA3 (2.23), KLHL5 (2.22), RNASE6 (2.22), TSPAN13 (2.2), TNFSF10 (2.19), MPEG1 (2.15), IL2RA (2.14), FGL2 (2.12), CD14 (2.11), CTSB (2.11), PTPN13 (2.1), TRAT1 (2.1), P2RY13 (2.09), PLCL1 (2.07), APP (2.07), NET1 (2.05), NEFL (2.01) | CD4 |
| CD4+ Memory T cells | TNFRSF4 (4.53), NPDC1 (4.45), PIEZO1 (4.37), FBXL8 (4.26), NEFL (4.22), PTGER2 (4.09), ST8SIA1 (4.05), LAIR2 (3.95), LYZ (3.93), MYO1F (3.92), LIMS1 (3.71), UTS2 (3.6), FLNA (3.57), GPR25 (3.56), CD300A (3.43), GDPD5 (3.38), MYBL1 (3.33), GLIPR2 (3.23), CRIP1 (3.18), MYO1G (3.18), CRIP2 (3.16), RNF214 (3.14), RAP1GAP2 (3.09), CHST7 (3.09), S100A4 (3.07), MAF (3.01), COTL1 (3), DIAPH2 (3), TRADD (2.98), IL32 (2.98), AQP3 (2.96), RAB37 (2.96), DUSP5 (2.89), CAPG (2.86), NBEAL2 (2.85), MFHAS1 (2.84), CORO1B (2.82), NCF4 (2.79), TRIB2 (2.77), CD99 (2.74), CDK2AP2 (2.74), CAPN2 (2.72), ST6GALNAC1 (2.7), SH3BP5 (2.7), INPP4B (2.7), YWHAH (2.69), TMEM116 (2.69), GNB2 (2.69), CX3CR1 (2.69), IFI44L (2.69), LY6E (2.68), PREX1 (2.66), LIME1 (2.66), SMCO4 (2.64), PKM (2.64), ITGA4 (2.62), RPS6KA1 (2.61), CTSZ (2.61), TSPO (2.56), CITED2 (2.54), SP140 (2.54), M6PR (2.53), SLC40A1 (2.51), GZMA (2.5), TLN1 (2.5), TRAV23DV6 (2.5), PPP1CA (2.48), MACF1 (2.48), CYB561 (2.47), PFN1 (2.47), LOXL1 (2.47), EMB (2.47), ZFP36L2 (2.47), CCR10 (2.46), FLI1 (2.44), CD226 (2.44), TRAPPC1 (2.43), NCALD (2.42), ANXA4 (2.42), PPDPF (2.4), RCSD1 (2.4), LY96 (2.39), ANXA6 (2.39), ITGAL (2.39), CAPNS1 (2.39), ZBP1 (2.38), STOM (2.38), JUND (2.38), IL10RA (2.38), LSR (2.37), PSTPIP1 (2.36), FERMT3 (2.36), MAP2K2 (2.36), ADAM8 (2.36), PRR7 (2.36), PRR5L (2.35), SIT1 (2.35), RNASE6 (2.35), P4HB (2.34), RNF126 (2.34), ITGB2 (2.33), CYBA (2.32), CCR2 (2.32), OAS1 (2.31), S100A10 (2.31), CD4 (2.3), FHL1 (2.3), EHBP1L1 (2.29), WNT10A (2.29), RAPGEF1 (2.29), CASP1 (2.28), JUNB (2.27), PPP1R18 (2.27), PRKCB (2.25), DUSP1 (2.24), MBOAT1 (2.24), ACVR1 (2.23), IL2RG (2.23), TSC2 (2.22), FAM50A (2.22), RGS19 (2.21), PLEC (2.21), PTPN13 (2.21), GBP4 (2.2), SNAI3 (2.19), GBP1 (2.19), TRPV2 (2.19), ARHGDIA (2.18), ATP2B4 (2.18), ARPC1B (2.18), CTSB (2.17), COX5A (2.17), RAB5C (2.17), PTPRCAP (2.16), MFSD10 (2.16), SELPLG (2.16), CLIC1 (2.16), AP2B1 (2.15), TNFSF10 (2.15), ZAP70 (2.14), SKAP1 (2.14), ANO9 (2.13), PTPN18 (2.13), CFL1 (2.13), ALYREF (2.13), TSPAN18 (2.13), CCDC92 (2.13), PHACTR2 (2.13), SASH3 (2.12), NRROS (2.11), IDH2 (2.11), KCNN4 (2.11), PBXIP1 (2.11), ATP8B2 (2.11), ARRB2 (2.1), POU2AF1 (2.1), MAN2B1 (2.1), S100A8 (2.1), TP53INP1 (2.1), MED15 (2.09), UBE2M (2.09), PGP (2.09), LSP1 (2.09), MYH9 (2.09), MVP (2.08), ZYX (2.08), SLC9A9 (2.08), DYNLL1 (2.08), TRAF1 (2.08), FLII (2.08), GALT (2.08), NDRG3 (2.07), CD82 (2.07), KANSL1-AS1 (2.07), APOBR (2.07), TPST2 (2.07), NOD2 (2.07), MAP3K1 (2.06), SYNGR2 (2.05), EHD1 (2.05), CYFIP1 (2.04), PTPN7 (2.04), LCP1 (2.04), WDR1 (2.04), PPP1R15A (2.04), MIF (2.04), ACTN4 (2.03), NUCB1 (2.03), TMEM200A (2.02), DOCK2 (2.02), OGDH (2.02), CD200R1 (2.02), CPNE2 (2.01), IER2 (2.01), PYCARD (2.01), SIGIRR (2.01), EXOSC6 (2), VASP (2), BIN2 (2) | CD4, CD45RO |
| CD4+ Naive T cells | ANKRD36BP2 (6.26), DACT1 (5.79), NOG (4.81), CTSL (4.59), PLAG1 (4.42), LRRN3 (3.95), SERPINE2 (3.88), GPR160 (3.73), RNF175 (3.7), CHI3L2 (3.68), EPPK1 (3.63), PLLP (3.6), EDAR (3.34), LEF1-AS1 (3.33), DSC1 (3.33), TMEM71 (3.27), CACHD1 (3.15), CDCA7L (3.15), NET1 (3.12), EPB41L3 (3.1), RNF144A (3.03), IGF1R (2.98), UBE2E2 (2.97), PXYLP1 (2.96), BTBD3 (2.92), PCSK5 (2.88), FHIT (2.8), MIR29C (2.78), EPHX2 (2.76), KRT72 (2.75), GSAP (2.71), APBA2 (2.69), SH3RF3 (2.67), COL18A1 (2.67), ATP10A (2.66), ARMCX2 (2.65), PTPRO (2.63), DEPDC7 (2.62), STAP1 (2.61), SCML4 (2.61), KCTD12 (2.57), MICU3 (2.56), IRS1 (2.49), ZNF285 (2.48), AK5 (2.46), VNN2 (2.45), MANSC1 (2.4), NUCB2 (2.39), HEMGN (2.38), HOOK1 (2.36), TRABD2A (2.36), MAN1C1 (2.35), RSAD2 (2.35), SIAH3 (2.35), RECK (2.33), KLHL29 (2.32), LTBP3 (2.32), ACTN1 (2.31), TSHZ2 (2.31), PAQR8 (2.29), WDR19 (2.29), ADTRP (2.27), RNF157-AS1 (2.25), PDK1 (2.25), ATM (2.25), CD7 (2.23), GIMAP1 (2.21), PLEKHB1 (2.21), RASGRF2 (2.19), CYSLTR1 (2.19), FAM184A (2.17), GAL3ST4 (2.17), ZNF514 (2.16), ZNF248 (2.14), SLC25A53 (2.14), IER3 (2.13), DPY19L4 (2.13), PDE7A (2.12), ZNF573 (2.11), TSEN2 (2.11), FAM216A (2.1), DPEP2 (2.09), ZNF175 (2.09), ZNF493 (2.08), PLXDC1 (2.08), TMEM263 (2.08), TMOD2 (2.05), ZNF879 (2.05), TCFL5 (2.04), ANKRD46 (2.04), CHMP7 (2.03), TMIGD2 (2.03), CERS6 (2.03), ZC3H6 (2.02), SPON1 (2.02), ZNF439 (2.02), CR2 (2.02), PHOSPHO2 (2.01), MEST (2.01), KBTBD11 (2.01), PTK2 (2.01), BBS2 (2) | CD4, CD45RA |
| CD8+ T cells | CD8B (9.58), CD8A (9.07), FGFBP2 (6.64), KLRD1 (6.44), KLRC4 (6.38), KLRC3 (6.3), GZMH (6.12), CRTAM (5.97), KLRF1 (5.71), NKG7 (5.65), CD160 (5.57), IGHV4-28 (5.52), CST7 (5.01), S100B (4.68), GNLY (4.66), F2R (4.64), TBX21 (4.6), EOMES (4.59), PROK2 (4.59), CTSW (4.58), MS4A1 (4.29), PRF1 (4.22), GZMB (4.16), SPON2 (4.07), CMC1 (3.9), ADRB2 (3.82), SLAMF7 (3.77), LAIR2 (3.7), SLCO4C1 (3.64), PRSS23 (3.61), UTS2 (3.53), PTGDR (3.52), CCL5 (3.42), SIGLEC17P (3.35), NCR3 (3.32), CACNA2D2 (3.28), CX3CR1 (3.23), CLIC3 (3.14), IL18RAP (3.09), TTC16 (3.08), TGFBR3 (3.08), IGHM (3), ADGRG1 (2.98), S1PR5 (2.95), GZMA (2.91), PLEK (2.9), CCL4 (2.9), PRR5L (2.84), IGKV4-1 (2.8), GZMK (2.8), CXCR6 (2.79), IFNG (2.79), LILRB1 (2.75), NR4A2 (2.74), KLRG1 (2.73), NT5E (2.71), ABCB1 (2.71), ITGAM (2.71), TTC38 (2.63), MYBL1 (2.63), LAG3 (2.62), RRAS2 (2.55), PATL2 (2.55), PPP2R2B (2.53), BANK1 (2.47), TPST2 (2.46), TMEM255A (2.42), B3GAT1 (2.42), SAMD3 (2.41), FCGBP (2.4), VPREB3 (2.4), HOPX (2.38), DUSP2 (2.37), ELANE (2.36), PTPRK (2.35), JAKMIP1 (2.31), FCRL3 (2.28), PDGFD (2.27), CXCR2 (2.26), LPCAT1 (2.26), TCL1A (2.25), SYTL2 (2.24), GAB3 (2.24), SETBP1 (2.21), ENPP5 (2.2), AZU1 (2.18), MAP3K8 (2.17), USP28 (2.16), ENPP4 (2.15), PRR5 (2.14), GALNT3 (2.13), PALLD (2.12), CCR5 (2.12), MATK (2.12), CD300A (2.08), MYOM2 (2.05), KIF21A (2.04), ZEB2 (2.03), A2M-AS1 (2.03), PTPN12 (2.03), IGKC (2.02) | CD8 |
| CD8+ Memory T cells | S100B (4.32), CA6 (4.31), FCER1G (3.69), KLRC4 (3.29), PPFIBP2 (3.13), ZEB1 (2.95), ERMN (2.89), FAM229B (2.89), CHKA (2.75), CD8B (2.71), BTBD11 (2.7), TCP11L2 (2.67), ZNF836 (2.66), CDC42 (2.65), ABCB1 (2.63), YBX3 (2.63), ZNF273 (2.56), YES1 (2.55), NRCAM (2.54), SNHG9 (2.53), WHAMM (2.52), SCML1 (2.5), DCTN6 (2.5), KLRC3 (2.36), ZNF667 (2.35), RAB33A (2.32), PGBD4 (2.27), SERPINF1 (2.24), TRAV30 (2.18), AIF1 (2.18), CXCL16 (2.17), RPPH1 (2.15), AK6 (2.15), COX14 (2.12), PET100 (2.11), CFAP20 (2.1), PTBP2 (2.09), RPA3 (2.08), SNORD8 (2.07), COX7C (2.06), SCARNA17 (2.03), KBTBD3 (2.02), ADPRM (2.02) | CD8, CD45RO |
| CD8+ Naive T cells | IFNG (4.85), CCL5 (4.66), CST7 (4.5), PMAIP1 (4.46), CA2 (4.44), NKG7 (4.4), IL2 (4.35), RGS2 (4.3), DUSP10 (4.29), SLC7A5 (4.27), OASL (4.23), KLRD1 (4.06), CMC1 (4.02), SAMSN1 (3.98), CREM (3.85), CCL4 (3.8), GPR15 (3.76), EOMES (3.74), MPZL3 (3.73), PELO (3.55), NR4A2 (3.46), EPHA4 (3.46), GNLY (3.46), GABARAPL1 (3.36), DUSP4 (3.26), LGALS1 (3.23), BHLHE40 (3.17), CXCL8 (3.1), DKK3 (3.1), PLXND1 (3.01), PDE4B (2.98), PTTG3P (2.97), CD58 (2.97), CTNNAL1 (2.96), GPR183 (2.93), GZMK (2.92), ENC1 (2.92), RGS1 (2.91), NR4A3 (2.91), RASD2 (2.9), TP53INP2 (2.88), ID2 (2.87), TXN (2.84), CD8A (2.78), CTSW (2.76), GYG1 (2.74), REL (2.74), TNF (2.73), QPCT (2.72), CCL20 (2.71), EGR2 (2.65), APOBEC3G (2.65), IL18RAP (2.64), FOSL2 (2.64), MXRA7 (2.62), TUBB6 (2.58), CMC2 (2.54), FAM83D (2.54), PPP1R16B (2.52), NECTIN3 (2.52), MCOLN2 (2.5), TAF13 (2.5), PTS (2.47), CXCR3 (2.45), APOM (2.43), CYSTM1 (2.42), CCNH (2.41), YIPF5 (2.39), SEC11C (2.39), F2R (2.39), ABCG1 (2.37), RNF19A (2.35), S100P (2.34), GGA2 (2.32), SH2D1A (2.29), PDE4D (2.29), TBX21 (2.27), IFIT2 (2.27), CDKN1A (2.26), PTTG1 (2.26), FTH1 (2.26), SLAMF7 (2.24), HSPA13 (2.24), GTF2IRD1 (2.23), JMJD6 (2.22), ALOX5AP (2.22), SKIL (2.22), TGFBR3 (2.21), SMAD7 (2.18), GPR65 (2.18), KIF21A (2.17), SRGN (2.16), PDE2A (2.16), SYTL2 (2.14), GZMH (2.14), ID3 (2.13), ULBP2 (2.13), CMSS1 (2.12), LINC-PINT (2.12), CSRNP1 (2.11), ZNF331 (2.11), SMIM4 (2.1), CGRRF1 (2.1), WHRN (2.09), MRPL10 (2.09), RHBDF2 (2.08), EAF2 (2.08), GNG2 (2.08), SUPT5H (2.07), TIGAR (2.07), ITGAV (2.05), SLA2 (2.03), CD40LG (2.03), SESN3 (2.03), SYNGR3 (2.02), TRMT10C (2.02), HEATR9 (2.02), SYAP1 (2.01) | CD8, CD45RA |
| Regulatory T cells | PMCH (8.59), FANK1 (8.55), CSF2RB (7.75), RTKN2 (6.55), KIF11 (6.04), RRM2 (6), ZC2HC1A (6), MEOX1 (5.94), FRMD4B (5.7), IL1R1 (5.69), P2RY14 (5.49), FAM110C (5.46), HLA-DQA1 (5.39), B3GALT2 (5.32), HLA-DQB1 (5.31), HACD1 (5.27), LGALS3 (5.24), MELK (5.17), GALNT3 (5.15), STAM (5.13), MIR155HG (5.11), DLGAP5 (4.95), IL1R2 (4.92), NABP1 (4.88), TSPAN13 (4.87), CCR5 (4.86), IL2RA (4.75), CEP55 (4.75), TOX (4.69), HPGD (4.54), SLC16A1 (4.51), FANCL (4.38), KIF15 (4.37), AURKA (4.31), INPP1 (4.26), ZNF80 (4.25), ATP1B1 (4.25), TIGIT (4.23), MTHFD2 (4.21), KIF18B (4.21), GNA15 (4.14), CXCR6 (4.13), SWAP70 (4.07), CDC20 (4.02), JAKMIP1 (3.99), GK (3.99), F5 (3.99), SHCBP1 (3.97), LAYN (3.95), NDC80 (3.92), TNFRSF1B (3.9), TOP2A (3.9), ZNF365 (3.9), CDCA7 (3.87), KIF14 (3.87), SYT4 (3.85), ZC3H12C (3.84), APOLD1 (3.82), LRRC2 (3.81), CCR6 (3.79), NEIL3 (3.77), CCNB1 (3.76), ZDBF2 (3.76), KIAA1841 (3.73), AHRR (3.73), MIB1 (3.72), ALDH8A1 (3.72), PRC1 (3.68), SLC1A4 (3.67), GPR19 (3.63), GINS1 (3.61), NUSAP1 (3.59), KLHL2 (3.58), SLC38A9 (3.58), ZNRF2 (3.55), MGST2 (3.55), ECT2 (3.54), ZC3H12D (3.53), CCDC141 (3.52), STIL (3.52), PIP5K1B (3.51), HMMR (3.51), SGMS1 (3.5), ACTA2 (3.46), BUB1B (3.46), SCG5 (3.44), IL18R1 (3.42), GJB6 (3.39), ZNF280C (3.38), CASP3 (3.38), KBTBD8 (3.37), MIAT (3.37), BARD1 (3.36), ASPM (3.35), NUF2 (3.32), ENTPD1 (3.31), IDH1 (3.31), RNF135 (3.3), DPY19L3 (3.29), RACGAP1 (3.28), EZH2 (3.27), SPIN4 (3.26), RCBTB1 (3.26), CCNB2 (3.24), STX7 (3.24), SRD5A1 (3.23), CPOX (3.22), PYHIN1 (3.21), LDLRAD4 (3.18), DERA (3.17), ESPL1 (3.16), RAP2A (3.15), VPS54 (3.15), FLVCR1 (3.15), PHTF1 (3.14), TBK1 (3.14), LCORL (3.13), TXNRD1 (3.12), MIR22HG (3.11), CD80 (3.09), ATXN1 (3.09), SDC4 (3.09), ATP10D (3.09), NCAPG (3.08), MYB (3.07), HDAC4 (3.04), FADD (3.02), BCL10 (3.02), CDC7 (3.01), CCNA2 (3), CYP51A1 (2.98), CMTM6 (2.98), ZNRF1 (2.97), SGO2 (2.97), TIGD2 (2.95), TNFRSF11A (2.94), LGMN (2.94), DPYSL2 (2.93), ICOS (2.89), HNRNPLL (2.89), CHSY1 (2.89), CKLF (2.88), HERPUD1 (2.86), CARD8-AS1 (2.84), HTATIP2 (2.84), SLCO4A1 (2.84), ZNF764 (2.83), TXNDC16 (2.83), FAM177A1 (2.82), TTF2 (2.82), ADAT2 (2.81), ACKR3 (2.8), EOGT (2.79), SERPINI1 (2.79), STK39 (2.79), CLDND1 (2.79), NETO2 (2.78), PSMA5 (2.78), HLA-DPA1 (2.77), WDR53 (2.77), SPTY2D1 (2.77), PLCL1 (2.77), EEF2K (2.76), SNX16 (2.76), RRM2B (2.76), E2F8 (2.75), SMC6 (2.75), METTL7A (2.75), RTN4IP1 (2.75), NAT1 (2.75), PRR11 (2.75), MGME1 (2.74), ORC6 (2.74), SLC39A10 (2.74), GLRX2 (2.73), KATNAL1 (2.73), SLC35F5 (2.73), CEP76 (2.73), KPNA2 (2.71), WDR3 (2.71), HLA-DRB1 (2.71), SLC35F3 (2.7), LTN1 (2.7), CCR3 (2.69), RAB11FIP1 (2.69), PCTP (2.69), NTAN1 (2.69), BAZ2B (2.69), CTLA4 (2.68), FAM174A (2.68), KLHL42 (2.68), FGL2 (2.66), FBXO45 (2.66), POC1B (2.65), PGAP1 (2.64), KLF10 (2.63), ABHD18 (2.63), ZBTB41 (2.63), RBBP8 (2.62), ZDHHC23 (2.62), MICB (2.62), AMMECR1L (2.62), DCP1B (2.61), LINC00467 (2.61), SGSH (2.61), HSF5 (2.6), ASPHD2 (2.6), WIPI1 (2.59), SLC35E3 (2.59), GLB1 (2.58), IKZF4 (2.58), SERAC1 (2.58), TRIM37 (2.58), OR7D2 (2.57), SLC16A10 (2.57), TMEM200B (2.57), AIM2 (2.56), MTM1 (2.56), AGBL2 (2.55), LAMP2 (2.55), GCA (2.55), WDR44 (2.55), IPCEF1 (2.54), HSPA1B (2.54), PROK2 (2.53), TTK (2.53), CRNKL1 (2.53), HSPA1A (2.53), RNF149 (2.52), PFKFB3 (2.51), PAM (2.51), SLC25A43 (2.51), PGM2 (2.51), AKR1C3 (2.5), SASS6 (2.49), RASGRP3 (2.49), BANP (2.48), SMC2 (2.47), PANK2 (2.47), TRIB1 (2.46), PKIA (2.46), SFT2D1 (2.45), IVNS1ABP (2.45), NSF (2.45), TRAK2 (2.45), GMNN (2.44), KDSR (2.44), LAMP3 (2.44), CCNE2 (2.44), FAM135A (2.44), HIBCH (2.43), UMAD1 (2.43), QTRT2 (2.42), DHFR2 (2.42), KCTD10 (2.41), KIF23 (2.41), TCP11L1 (2.41), DNAJC9 (2.4), CLHC1 (2.4), SAMD9L (2.39), TMEM65 (2.39), TMEM169 (2.39), CCNG2 (2.39), FUCA2 (2.39), KIF20A (2.39), CHST11 (2.38), LAX1 (2.38), GPR137B (2.38), SLC16A6 (2.38), HMGB2 (2.38), ACYP1 (2.38), RAD51AP1 (2.38), CALHM2 (2.38), CBR4 (2.38), BTN2A2 (2.37), KAT2B (2.37), MREG (2.37), ZNF267 (2.37), ARAP2 (2.37), FAM13B (2.37), NCAPH (2.36), PARP12 (2.36), ZNF564 (2.36), TMEM154 (2.36), SKAP2 (2.35), ADAM12 (2.35), DEPDC1B (2.35), SNX10 (2.35), ST8SIA4 (2.35), FASTKD1 (2.35), ATP6V1D (2.35), BLM (2.34), C2CD5 (2.34), CENPA (2.33), NAA30 (2.33), YWHAG (2.33), HELLS (2.33), STAMBPL1 (2.33), PAOX (2.33), GPSM2 (2.32), TRIP13 (2.32), MYO5A (2.32), CARD6 (2.32), SPOPL (2.31), SETD7 (2.31), HCCS (2.31), SLC25A44 (2.31), DAAM1 (2.3), CENPF (2.3), ZWINT (2.3), KIF1B (2.29), IL17RB (2.29), TOX2 (2.29), HMGCR (2.29), PPM1B (2.29), DCLRE1A (2.28), CCDC77 (2.28), ISOC1 (2.28), FBXO30 (2.27), RFXAP (2.27), RMI1 (2.27), TAF5 (2.27), BCAS1 (2.26), TMEM14A (2.26), SRXN1 (2.26), ZFC3H1 (2.26), IMPA1 (2.26), BCL6 (2.26), NDRG1 (2.26), PERP (2.25), THAP6 (2.25), SACS (2.25), B4GALT5 (2.25), ZNF367 (2.24), NCOA3 (2.23), TEFM (2.23), TMEM135 (2.22), PSD3 (2.21), NLRC3 (2.21), IKZF2 (2.2), GPR155 (2.2), ALG2 (2.2), UBR1 (2.2), KBTBD6 (2.2), ARMT1 (2.2), EPM2AIP1 (2.19), SNX4 (2.19), HLA-DRA (2.19), FEZ2 (2.19), PPP1R26 (2.18), INPP5F (2.18), SLC35A1 (2.17), B3GNTL1 (2.17), EPSTI1 (2.17), DGKE (2.16), MAP3K5 (2.16), TTC13 (2.16), SP2 (2.16), PRMT3 (2.16), SEPHS2 (2.15), RHOXF1 (2.15), DUSP16 (2.15), TTL (2.15), ZBTB20-AS1 (2.14), NDUFAF1 (2.14), EIF4ENIF1 (2.13), SLC19A2 (2.13), KLHL28 (2.13), PSPH (2.13), VPS4B (2.13), ANXA2 (2.12), CENPM (2.12), IQGAP2 (2.12), HS3ST3B1 (2.12), SLC35B3 (2.12), INTS2 (2.11), ABAT (2.11), DCAF12 (2.11), CCL20 (2.11), PPP4R1 (2.1), PEX13 (2.1), TSPAN5 (2.1), PKD2 (2.09), UBA3 (2.09), LOXL1-AS1 (2.09), LRP8 (2.09), SLFN11 (2.09), HSPBAP1 (2.08), IKBIP (2.08), CHUK (2.08), DDX60L (2.07), ADIPOR2 (2.07), GBE1 (2.07), BATF (2.06), DHX29 (2.06), SNORA25 (2.06), SLF2 (2.06), SHMT2 (2.05), CEP97 (2.05), SLC25A17 (2.05), NCF2 (2.05), SIDT2 (2.05), GALNT7 (2.04), NOC3L (2.04), NINJ2 (2.04), UNC5CL (2.04), DOCK11 (2.03), PDIK1L (2.03), RNF145 (2.03), CTSH (2.03), SETDB2 (2.03), PUS7L (2.03), SAPCD2 (2.03), LBR (2.03), ATG2B (2.03), PCMTD1 (2.03), ZNF420 (2.03), VPS13C (2.03), HERC2 (2.02), LINC00888 (2.02), HACE1 (2.01), HPSE (2.01), PHKB (2.01), CHCHD4 (2.01), IBTK (2.01), SLC37A3 (2.01), UBTD2 (2.01), GSKIP (2.01), FAM118B (2), ATXN3 (2), AP1AR (2), KLHL34 (2) | CD4, CD25 |

**Supplementary Table 2. The gene sets and CITE-seq markers used for T cell subtyping.** For each gene, the value following it in parentheses is its gene weight. The CITE-seq markers selected reflect canonical markers of each T cell subtype [(Zheng *et al.*, 2017; Rausser *et al.*, 2021)](https://www.zotero.org/google-docs/?Blas25).

**References**

[10k Cells from a MALT Tumor (2018) Single Cell Gene Expression Dataset by Cell Ranger 3.0.0. *10x Genomics*.](https://www.zotero.org/google-docs/?O8VEIl)

[Aran,D. *et al.* (2019) Reference-based analysis of lung single-cell sequencing reveals a transitional profibrotic macrophage. *Nat. Immunol.*, **20**, 163–172.](https://www.zotero.org/google-docs/?O8VEIl)

[Blondel,V.D. *et al.* (2008) Fast unfolding of communities in large networks. *J. Stat. Mech. Theory Exp.*, **2008**, P10008.](https://www.zotero.org/google-docs/?O8VEIl)

[De Biasi,S. *et al.* (2020) Marked T cell activation, senescence, exhaustion and skewing towards TH17 in patients with COVID-19 pneumonia. *Nat. Commun.*, **11**, 3434.](https://www.zotero.org/google-docs/?O8VEIl)

[Frost,H.R. (2020) Variance-adjusted Mahalanobis (VAM): a fast and accurate method for cell-specific gene set scoring. *Nucleic Acids Res.*, **48**, e94–e94.](https://www.zotero.org/google-docs/?O8VEIl)

[Hao,Y. *et al.* (2021) Integrated analysis of multimodal single-cell data. *Cell*, **184**, 3573-3587.e29.](https://www.zotero.org/google-docs/?O8VEIl)

[Li,L. *et al.* (2021) Improved integration of single-cell transcriptome and surface protein expression by LinQ-View. *Cell Rep. Methods*, **1**, 100056.](https://www.zotero.org/google-docs/?O8VEIl)

[Liberzon,A. *et al.* (2011) Molecular signatures database (MSigDB) 3.0. *Bioinformatics*, **27**, 1739–1740.](https://www.zotero.org/google-docs/?O8VEIl)

[Mabbott,N.A. *et al.* (2013) An expression atlas of human primary cells: inference of gene function from coexpression networks. *BMC Genomics*, **14**, 632.](https://www.zotero.org/google-docs/?O8VEIl)

[McInnes,L. *et al.* (2018) UMAP: Uniform Manifold Approximation and Projection. *J. Open Source Softw.*, **3**, 861.](https://www.zotero.org/google-docs/?O8VEIl)

[R Core Team R: A Language and Environment for Statistical Computing.](https://www.zotero.org/google-docs/?O8VEIl)

[Rausser,S. *et al.* (2021) Mitochondrial phenotypes in purified human immune cell subtypes and cell mixtures. *eLife*, **10**, e70899.](https://www.zotero.org/google-docs/?O8VEIl)

[Robinson,M.D. *et al.* (2010) edgeR: a Bioconductor package for differential expression analysis of digital gene expression data. *Bioinformatics*, **26**, 139–140.](https://www.zotero.org/google-docs/?O8VEIl)

[Satija,R. *et al.* (2015) Spatial reconstruction of single-cell gene expression data. *Nat. Biotechnol.*, **33**, 495–502.](https://www.zotero.org/google-docs/?O8VEIl)

[Schiebout,C. and Frost,H.R. (2022) CAMML: Multi-Label Immune Cell-Typing and Stemness Analysis for Single-Cell RNA-sequencing. *Pac. Symp. Biocomput.*](https://www.zotero.org/google-docs/?O8VEIl)

[Zhang,Z. *et al.* (2019) SCINA: A Semi-Supervised Subtyping Algorithm of Single Cells and Bulk Samples. *Genes*, **10**, 531.](https://www.zotero.org/google-docs/?O8VEIl)

[Zheng,G.X.Y. *et al.* (2017) Massively parallel digital transcriptional profiling of single cells. *Nat. Commun.*, **8**, 14049.](https://www.zotero.org/google-docs/?O8VEIl)
